# The Peroxygenase Activity of the *Aspergillus flavus* Caleosin, AfPXG, Modulates the Biosynthesis of Aflatoxins and Their Trafficking and Extracellular Secretion via Lipid Droplets

**DOI:** 10.3389/fmicb.2018.00158

**Published:** 2018-02-06

**Authors:** Abdulsamie Hanano, Mari Alkara, Ibrahem Almousally, Mouhnad Shaban, Farzana Rahman, Mehedi Hassan, Denis J. Murphy

**Affiliations:** ^1^Department of Molecular Biology and Biotechnology, Atomic Energy Commission of Syria, Damascus, Syria; ^2^Genomics and Computational Biology Research Group, University of South Wales, Pontypridd, United Kingdom

**Keywords:** lipid droplets, caleosin, *Aspergillus flavus*, peroxygenase, aflatoxin

## Abstract

Aflatoxins (AF) are highly detrimental to human and animal health. We recently demonstrated that the *Aspergillus flavus* caleosin, AfPXG, had peroxygenase activity and mediated fungal development and AF accumulation. We now report the characterization of an *AfPXG*-deficient line using reference strain NRRL3357. The resulting fungal phenotype included a severe decrease in mycelium growth, failure to sporulate, and reduced AF production. Increasing cellular oxidative status by administration of hydrogen peroxide and cumene hydroperoxide did not restore the *AfPXG*-deficient phenotype, which suggests that *AfPXG*-deficiency is not directly related to oxidative stress. To investigate possible alternative roles of *AfPXG*, a gain of function approach was used to overexpress *AfPXG*, with the reporter gene *Gfp*, in an *AfPXG*-deficient line, termed *AfPXG^+^*. The resulting phenotype included elevated numbers of stable lipid droplets (LDs) plus enhanced AF production. Highly purified LDs from *AfPXG^+^* cultures sequestered AF and this ability was positively correlated with overall LD number. Site-specific mutagenesis of *AfPXG* to delete Histidine 85 (AfPXG_His85_), a residue essential for its catalytic activity, or deletion of the putative LD targeting domain (AfPXG_D126-140_), showed that AfPXG-peroxygenase activity was required for AF biosynthesis and that integration of AF into LDs was required for their export via a LD-dependent pathway. Ectopic expression in fungal cells of the plant LD-associated protein, oleosin, also resulted in both additional LD accumulation and enhanced AF secretion. These results suggest that both fungal LDs and their associated caleosin proteins are intimately involved in the biosynthesis, trafficking, and secretion of AF.

## Introduction

The aflatoxins (AF) are a group of lipid-derived furanocoumarin mycotoxins exhibiting both acute and chronic toxicity in humans due to contamination of fresh and stored food products by certain ascomycete fungi, most notably *Aspergillus flavus* and *Aspergillus parasiticus* (Yu et al., 2004; Shephard, 2008; Yu, 2012). Other AF-producing fungi include *Aspergillus bombycis*, *Aspergillus nomius*, *Aspergillus ochraceoroseus*, and *Aspergillus pseudotamarii* (Yu et al., 2004), plus the anamorph, *Emericella venezuelensis* (Frisvad and Samson, 2004). In terms of toxicity to humans, the most widespread and important form of AF is aflatoxin B_1_, [(6aR,9aS)-2,3,6a,9a-Tetrahydro-4-methoxy-1H,11H-cyclopenta[c]furo[3′,2′:4,5]furo[2,3-h][1]benzopyran-1,11-dione]. Aflatoxin B_1_ (AFB1) can be present either during and/or after fungal growth on a food product and its ingestion can result in acute and often fatal poisoning of humans and livestock species. In terms of chronic exposure, AFB1 is regarded as the most potent environmental carcinogen identified to date, with hepatocellular carcinoma as a major risk factor (Yu et al., 2004; Yu, 2012).

AF are ultimately synthesized from acetyl-CoA via fatty acid, polyketide, and xanthone intermediates in a complex pathway that, in the case of *A. parasiticus*, is encoded by a cluster of 25 physically linked genes that are coordinately regulated (Yu et al., 2004; Keller et al., 2005). It is estimated that as much as 90% of total synthesized AF can be secreted from fungal cells. Therefore, the processes of AF metabolism, trafficking, and secretion involve a complex series of biosynthetic, sequestration, and export pathways involving several subcellular compartments (Roze et al., 2007). The initial stages of AF biosynthesis, from acetyl-CoA to the polyketide norsolorinic acid, probably occur in peroxisomes (Maggio-Hall et al., 2005; Roze et al., 2007) with subsequent steps within the cytosol, vacuoles, and small vesicle-like structures (Hong and Linz, 2008, 2009). Vesicles play key roles in AF metabolism, transport, and eventual secretion and the term “aflatoxisome” has been coined to describe the population of small (<2 μum diameter) vesicles that carry out post-polyketide biosynthetic reactions that result in the accumulation of end products, such as AFB1 (Chanda et al., 2009). Aflatoxisomes, which are at least partially derived from the ER–Golgi–endosome endomembrane system, are loaded with AF biosynthetic enzymes synthesized either on free ribosomes or via the ER–Golgi (Roze et al., 2007) and with norsolorinic acid synthesized by peroxisomes (Chanda et al., 2009; Kistler and Broz, 2015).

The subsequent fate of mature AF-loaded aflatoxisome vesicles has yet to be fully resolved but it is proposed that aflatoxisomes may release their cargoes via exocytosis, with a possible second trafficking pathway whereby the vesicles fuse with vacuoles, which would result in a turnover and recycling of their enzyme and AF cargoes (Linz et al., 2014). Inhibition of the latter pathway, as seen in *avaA* mutants and following Sortin3 treatment, would then greatly accelerate AF synthesis and export as part of a rapid and potent response to environmental stimuli (Ehrlich et al., 2012; Linz et al., 2014; Kistler and Broz, 2015). The precise mechanism of AF secretion, whether via conventional exocytosis or via one of several alternative Golgi-independent pathways, remains to be determined (Shoji et al., 2014; Kistler and Broz, 2015).

AF biosynthesis in *A. flavus* and *A. parasiticus* is upregulated in response to a variety of endogenous and exogenous environmental cues especially oxidative stress and the presence of reactive oxygen species (ROS; Reverberi et al., 2012). Indeed, oxidative stress may be a prerequisite for AF production (Jayashree and Subramanyam, 2000; Fountain et al., 2014). It is proposed that, in response to external stimuli including extracellular ROS, NADPH oxidase A (NoxA) initiates a primary burst of intracellular ROS that activates the master regulator gene, AflR, which in turn stimulates expression of the AF pathway genes and biosynthesis of AF (Roze et al., 2007). In addition, several intermediates in the AF biosynthetic pathway, which includes no fewer than seven P450 monooxygenases, are capable of generating secondary releases of ROS that may be involved with the latter stages of AF secretion from fungal cells (Roze et al., 2015). Therefore, the biological roles of AF in fungi may be at least partially related to oxidative stress response/tolerance and antioxidant protection (Fountain et al., 2014).

The stimulation of AF biosynthesis and secretion is not necessarily a direct response to external ROS. For example, endogenous lipids that are induced following oxidative stress, such as unsaturated fatty acids, oxylipins such as hydroperoxides, and volatiles such as 2-ethyl-1-hexanol, can significantly modulate the biosynthesis of AF and other mycotoxins (Roze et al., 2007; Gao and Kolomiets, 2009; Brodhun and Feussner, 2011; Fountain et al., 2014). The precise nature of the oxidant(s) can also be important so, in both *A. flavus* and *A. parasiticus*, AF biosynthesis in the fungus was inhibited by 13-hydroperoxides produced by an infected plant, but 9-hydroperoxides had no such effect (Burow et al., 1997). In addition to their effects on AF metabolism, oxylipins and other fungal lipids are implicated in the regulation of numerous other aspects of development including spore formation and germination as well as in various aspects of fungal/plant pathogenesis interactions and crosstalk (Fanelli and Fabbri, 1989; Doehlert et al., 1993; Calvo et al., 2001; Brodhagen et al., 2007; Tsitsigiannis and Keller, 2007).

In recent years, it has been recognized that, in addition to acting as long-term carbon stores, subcellular lipid droplets (LDs) in fungi and other organisms have numerous active roles in metabolism, trafficking, and stress responses (Murphy, 2012; Radulovic et al., 2013; Chang et al., 2015; Fan et al., 2015; Shimada et al., 2015; Ortiz-Urquiza, 2016). Among other functions fungal LDs have been shown to sequester toxins (Chang et al., 2015), generate oxylipins (Shimada et al., 2015), and mediate virulence (Ortiz-Urquiza, 2016). Another emerging factor is the role of a wide range of LD-associated proteins that appear to play crucial roles in many aspects of fungal development. Such proteins include caleosins (Fan et al., 2015; Hanano et al., 2015), Vip1p (Meyers et al., 2017), PpoA (Tsitsigiannis et al., 2004), and several endomembrane trafficking components including Rab7-like Ypt7p (Bouchez et al., 2015).

We have recently shown that the peroxygenase responsible for the metabolism of hydroperoxides in *A. flavus* is a member of the caleosin gene family that is found in the majority of currently sequenced fungal genomes and is also ubiquitous in all sequenced plant genomes (Murphy, 2012; Hanano et al., 2015). Deletion of this gene in *A. flavus* prevented fungal development while partial silencing reduced fungal growth and formation of conidia while several AF biosynthetic genes were downregulated and AF production was reduced by <40-fold (Hanano et al., 2015). A key role for caleosins in AF metabolism is also suggested by the downregulation of the *A. parasiticus* caleosin gene in non-AF producing mutants (Wilkinson et al., 2011). In this study, we used gene knockouts and site-specific mutagenesis to elucidate the role of the *A. flavus* caleosin in AF biosynthesis and secretion, plus the related roles of LD formation and trafficking in these processes. The results implicate both caleosins and LDs in the induction and processing of AF, and suggest that they play important and previously unrecognized roles in the trafficking and secretion of AF, alongside the established aflatoxisome pathway.

## Materials and Methods

### Bioinformatics Analysis/Gene Characterization

For sequence retrieval and identification of caleosin in fungal species, putative CLO sequences of *A. flavus* (AflCLO), *Erysiphe necator* (EnCLO), *Neurospora crassa* (NcCLO), *Magnaporthe oryzae* (MoCLO), *Beauveria bassiana* (BbCLO), *Ustilago maydis* (UmCLO), *Rhodotorula toruloides* (RtCLO), *Gonapodya prolifera* (GprCLO), *Rhizophagus irregularis* (RiCLO), *Allomyces macrogynus* (AmaCLO), *Rozella allomycis* (RaCLO) were obtained from NCBI (http://www.ncbi.nlm.nih.gov/) via local BLAST+ searches (Chen et al., 2015) and analyzed as described in Supplementary Materials.

### Materials, Chemicals, Strains, Culture Conditions, and Treatments

Oligonucleotides were purchased from either Eurofins or Sigma-France. Aniline, thiobenzamide, cumene hydroperoxide (Cu-OOH), and aflatoxins B1 were purchased from Sigma–Aldrich, Germany. [1-^14^C] Oleic acid (52 μCi mmol^-1^) was purchased from PerkinElmer Life Sciences. Names, source, genetic, and biochemical characteristics of all strains and lines that used in this study are summarized in **Table 1**. Stock cultures of *A. flavus* were maintained in slant tubes at 4°C on potato dextrose agar (PDA) (Difco Laboratories, United States). For solid or liquid cultures of *A. flavus*, stock cultures were transferred onto Petri dishes containing PDA or into a 500-mL Erlenmeyer flask containing 100 μL of PD broth and allowed to develop for 7 days at 28°C. To ensure an effective induction of the *Adh1* promoter, the *AfPXG*-overexpressed line was cultured in YEPE medium containing 1% yeast extract, 2% peptone (Difco Laboratories, United States) and 2% ethanol and 2% Bacto-agar (Difco Laboratories, United States) was used to solidify the medium (Ruohonen et al., 1995). Treatments with hydrogen peroxide (H_2_O_2_) (100 and 200 μM) or with Cu-OOH (25 and 50 μM) were performed on fresh cultures of *A. flavus* prepared overnight in 5 mL PD broth. Next, 100 μL samples of treated and untreated cultures were cultured from a single inoculation point on Petri dishes containing PDA and incubated at 28°C for 4 days.

**Table 1:** Strains and lines of *Aspergillus flavus* used in this study.

Strain/lines nomination	Mother strain	Genetic manipulation	PXG enzymatic activity	Lipid droplets accumulation	Aflatoxin B1 biosynthesis/secretion
*Af/Wt*	*A. flavus* Wt^a^	*Non*	++	+ (only at T0)	+/+
*AfPXG null*	*A. flavus* Wt	*PXG knock out*	-	-	-/-
*siRNAPXG*	*A. flavus* Wt	*PXG knock down*	+		
*AfPXG+*	*A. flavus* Wt	*PXG Overexpression*	+++	++	++/++
*AfPXG_His85_*	*A. flavus* Wt	*PXG mutant His 85*	-	+	-/-
AfPXG_D126-140_	*A. flavus* Wt	*PXG deletion 126–140*	+	-	+/-
*OLE1/AfWt*	*A. flavus* Wt	*OLE1 expression*	++	++	+/++
*OLE1/PXGΔ*	*AfPXG null*	*OLE1 expression*	-	++	-/-
*Yeast/Wt*	*S. cerevisiae* Wa6^b^	*Non*	-	-	NA^c^
*Yeast/AfPXG_Wt_*	*S. cerevisiae* Wa6	*PXG_WT_ expression*	+++	++	NA
*Yeast/AfPXG_His85_*	*S. cerevisiae* Wa6	*PXG_His85_ expression*	-	++	NA
*Yeast/AfPXG_D126-140_*	*S. cerevisiae* Wa6	*PXG_D126-140_ expression*	++	-	NA

^a^*Aspergillus flavus NRRL3357. This strain was kindly supplied by Prof. Marie-Laure Fauconnier, from the faculty of Agricultural Sciences, Gembloux, Belgium.*

*^b^Saccharomyces cerevisiae Wa6 was previously obtained from the Institute of Plant Molecular Biology, Strasbourg, France. ^*c*^Not applicable*.

### Replacement and siRNA-Silencing of *AfPXG* Gene

The replacement of the *AfPXG* gene by the hygromycin-resistance gene (*Hyg^r^*) in the genome of *A. flavus* NRRL3357 was performed by fusion PCR as recently described (Hanano et al., 2015). More details are available in Supplementary Materials. Delivery of siRNA to protoplasts was performed in sterile 1.5 mL tubes. Ten microliters of each siRNA primer (100 nM) was mixed with an equal volume of Lipofectin reagent (Invitrogen Life Technologies, United Kingdom) and allowed to stand for 15 min at 20°C. A volume of 50 μL of protoplasts was added, gently mixed, and incubated at 20°C for 24 h to allow transfection to proceed (Whisson et al., 2005). The transfected protoplasts were inoculated in 10 mL of PD medium with 1.2 M of sorbitol for 7 days at 28°C in the dark. A similar treatment of protoplasts without siRNA or with non-specific siRNACt was performed as negative controls. All experiments were performed out using three biological replicates.

### Overexpression of *AfPXG* or OLE1

Overexpression of *A. flavus* peroxygenase-encoding gene (*AfPXG*) was carried out under the control of *AdhI* promoter using *Gfp* as a reporter gene. The construct *AdhI/AfPXG/Gfp* was introduced into the genome of the AfPXGΔ. Methods of expression are detailed in Supplementary Figure S2.

### Generation of Mutated AfPXG and Expression of the Recombinant Protein

Variants of *AfPXG* were generated using the recombinant plasmid pVT102U/*AfPXG* (Hanano et al., 2015). All primers used in this section are listed in Supplementary Table S3. The replacement of Histidine 85, a residue essential for PXG catalytic activity, by valine, was performed by a site-directed mutagenesis approach as described previously (Hanano et al., 2006). The modified codon is indicated in bold in the primers used H85/VF and H85/VR. Deletion of the transmembrane domain of AfPXG located between residues 126 and 140 was done by a PCR-based strategy (Kamper, 2004) using primers (D126–140F and D126–140R). The verified sequences of the pVT102U*/AfPXG* variants were then introduced into *Saccharomyces cerevisiae* Wa6 (*ade*, *his7-2 leu2-3 leu2-112 ura3-52*) (Schiestl and Gietz, 1989). Expression of the recombinant AfPXG in transformed yeast cells and the isolation of the respective subcellular fractions was carried out as described previously (Hanano et al., 2006). The modified genes were introduced into the strain AfPXGΔ as described before.

### Biomass and Conidia Number Measurements

Fungal biomass was determined as described previously (Hanano et al., 2015). Mycelium dry weights were subsequently evaluated according to Rasooli and Razzaghi-Abyaneh (2004). See Supplementary Materials.

### Preparation of *A. flavus* Subcellular Fractions and Peroxygenase Activities Assay

Isolation of microsomal and LD fractions from fungal cells was performed essentially as described by Record et al. (1998) and Ferreira de Oliveira et al. (2010) with brief modification as described previously (Hanano et al., 2015) (see Supplementary Materials).

### Analysis of LDs

Microscopic imaging was performed at a magnification of 40× under a LEICA MPS60 microscope using an Olympus FE-4000 camera. The purity of LD preparation, their native encapsulation and their number per milliliter were evaluated by a Flow cytometer (BD FACSCALIBUR, Biosciences, United States). LD size distributions (% frequency) were determined using a laser granulometer (Malvern Mastersizer S; Malvern Instruments, United Kingdom) fitted with a 320 mm lens as described previously (White et al., 2006).

### SDS-PAGE, Western Blotting, and Surface Immunofluorescence

LD-associated proteins were isolated according to Katavic et al. (2006) and analyzed by SDS-PAGE then immunodetected by incubating the membrane with a polyclonal antibody prepared from the complete sequence of the CLO1 caleosin isoform from *Arabidopsis thaliana*, as described previously (Hanano et al., 2006, 2016). Surface immunofluorescence was performed according to Chanda et al. (2010) where rabbit antibodies against aflatoxin B1 (Sigma–Aldrich, United States) were used as primary antibodies to detect AFB1 on the mycelium surface. Fluorescein isothiocyanate (FITC)-labeled anti-rabbit IgG (Sigma–Aldrich, United States) was used as a secondary antibody.

### Genes, Primers, and Transcripts Analysis

Supplementary Table S1 summarizes the gene order, NCBI-accession number, gene name, enzyme name, and the respective catalytic activity for the *A. flavus* NRRL3357 AF biosynthesis cluster genes. Nucleotide sequences of primers used in this section are listed in Supplementary Table S2 and method is explained in Supplementary Materials.

### Extraction, Clean-Up, TLC, and HPLC Analysis of Aflatoxin

The extraction of AF was carried out according to Bertuzzi et al. (2011) using 100 mL of chloroform for 1 h on a rotary-shaker and extracts were purified as described previously (Shannon et al., 1983). More details are available in the Supplementary Materials.

### Statistics

All data presented were expressed as means ± standard deviation. Comparisons between control and treatments were evaluated by *t*-test. Difference from control was considered significant as *P* < 0.05, very significant as *P* < 0.01, and highly significant as *P* < 0.001.

## Results

### Bioinformatics Analysis of Caleosin Genes from *A. flavus* and Other Fungi

**FIGURE 1:**
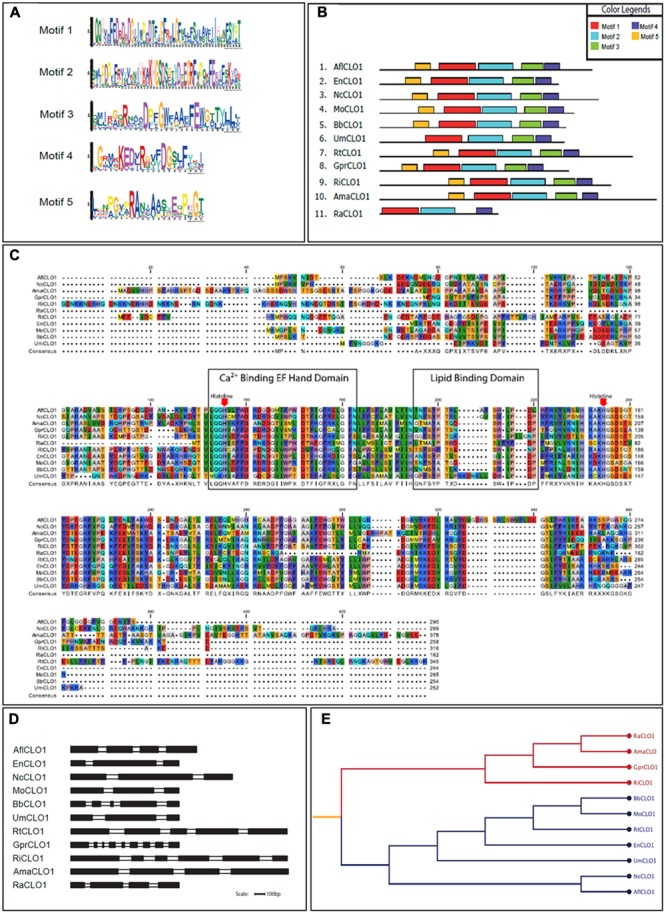
Bioinformatics analysis of caleosin sequences derived from the genomes of *A. flavus* and other representative fungal species. **(A)** Motif sequences generated from fungal caleosin protein sequences using http://meme-suite.org/. **(B)** Block representations of the location on caleosin sequences of the five predicted motifs shown in panel **(A)**. Each motif is color coded as shown in the upper right box. **(C)** Amino acid multiple sequence alignment of caleosins from selected fungal species. Multiple alignments were performed using Clustal Omega (ClustalO) from https://www.ebi.ac.uk/Tools/msa/clustalo/. The CLC sequence viewer from CLCBio was used to inspect the alignments. Caleosins were obtained from the NCBI database (http://www.ncbi.nlm.nih.gov) under accession numbers presented in Supplementary Table S4. Domain properties of the proteins were analyzed using the UniProt platform (http://www.uniprot.org). Color coding is as follows: the multiple sequence alignment residues are colored according to Rasmol amino color scheme, first boxed residues indicate the combined Ca^2+^ binding EF hand domain and second boxed residues indicate the lipid binding domain. The invariant heme-binding histidine motif is marked in red with down arrows. **(D)** Intron/exon structures of selected fungal caleosin genes generated using WebScipio. Exons are depicted as solid bars while introns are clear bars. Scale bar is 100 bp. **(E)** Phylogenetic tree to represent the cladistic relationship between *A. flavus* and other representative fungal species. Sequences from the more advanced Dikarya species, including *A. flavus*, are shown in blue and sequences from the more basal and divergent taxa are shown in red.

From a survey of the more than 300 currently available fungal genomes in the NCBI and individual fungal databases, it was found that there was typically one, or occasionally two, caleosin-like sequence(s) in most species (data not shown). This included all major fungal taxa, namely the Dikarya (i.e., Basidiomycota plus Ascomycota), Zygomycota, Chytridiomycota, Blastocladiomycota, and Glomeromycota (James et al., 2006). Caleosin-like sequences were also present in some of the few published genomes from more basal or derived fungal clades, such as Cryptomycota and Microsporidia (Supplementary Table S4). In some cases there were multiple caleosin-like entries but many of these were partial sequences and/or lacked essential canonical domains and were therefore not included in the analysis. In **Figures 1A–D**, the protein motif, alignment, gene structure (intron/exon organization) and phylogeny analyses of 11 selected caleosin sequences is presented. Phylogenetic analyses of the 11 selected sequences (see cladogram in **Figure 1E**) shows that all the Dikarya sequences (five Ascomycota and two Basidiomycota) clustered in a single clade that was well separated from the three less closely related divisions, Chytridiomycota, Blastocladiomycota, and Glomeromycota, and from the basal fungal division, Cryptomycota. Intron/exon organization data (**Figure 1D** and Supplementary Table S4) show that caleosin gene organization is relatively divergent in fungi, as has also been noted in plants (Song et al., 2014). The predicted protein sequences showed high levels of identity or conservative substitution, especially in the more closely related Dikarya taxa. As expected, caleosins in the less closely related and more basal taxa were less similar, but even here there was strong sequence conservation in the key motifs that include the canonical EF-hand, heme-binding and lipid-binding domains.

### Exogenous Oxidative Status Does Not Restore the *AfPXG*-Deficient Phenotype

**FIGURE 2:**
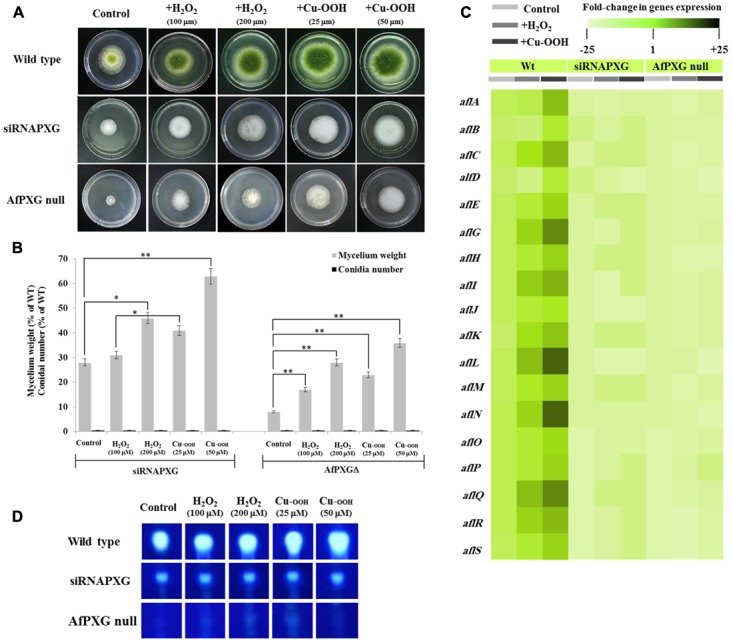
Increasing oxidative status does not restore the *AfPXG*-mutant phenotype. **(A)** Effect of H_2_O_2_ and cumene hydroperoxide (Cu-OOH) on the morphology of fungal phenotypes grown on PDA plates for 4 days at 28°C. Fungal growth was incubated with H_2_O_2_ and Cu-OOH at the indicated concentration. **(B)** Evaluation of fungal biomass (light-gray columns) and conidia number (black columns) for both lines, siRNAPXG2 and AfPXGΔ. Fungal biomass from each of the *AfPXG*-deficient lines exposed to H_2_O_2_ or Cu-OOH was significantly different from those of non-treated lines when analyzed by *t*-test (^∗^*P* < 0.05; ^∗∗^*P* < 0.01). **(C)** Relative quantification of transcript levels of AFB1-biosynthesis genes in *AfPXG*-deficient lines as a function of treatments with H_2_O_2_ (200 mM) or Cu-OOH (50 mM) compared with controls. For each gene, the transcript level was evaluated by qRT-PCR as described in Section “Materials and Methods.” Three measurements were taken in three cDNAs prepared from three individual fungal growth for each treatment. The color scale (white–green–black) indicates relative changes of transcript abundance of –25-, 1-, and +25-fold, respectively. For each gene, the expression level in WT unexposed to H_2_O_2_ or Cu-OOH was defined as 1, and the corresponding abundance changes under treatments were calculated directly using software installed in the Applied Biosystems qPCR system. **(D)** TLC-analysis of AFB1 produced by *AfPXG*-deficient lines as a function of treatment with H_2_O_2_ or Cu-OOH compared with controls.

We previously reported that silencing of *AfPXG* enhances ROS-degrading enzyme activities suggesting that the downregulation of AF biosynthesis in *AfPXG*-silenced strain takes place via neutralizing the oxidative status (Hanano et al., 2015). Unexpectedly, treatment of siRNAfPXG and AfPXGΔ with various concentrations of H_2_O_2_ or Cu-OOH, two compounds known to enhance the oxidative stress and therefore induce the production of AF in *A. flavus* (Emri et al., 2015), doubled vegetative growth in both lines but did not remedy their deficiency in sporulation (**Figures 2A,B**). Moreover, the administration of H_2_O_2_ or Cu-OOH on siRNAfPXG and AfPXGΔ did not modify transcript levels of AF biosynthesis-cluster genes in both lines, compared with WT controls (**Figure 2C**). This was confirmed by a rapid TLC-screening for AF secreted in the culture medium of siRNAfPXG and AfPXGΔ compared with WT (**Figure 2D**). These results indicate that enhancement of oxidative status in the *AfPXG*-deficient lines did not restore their respective phenotypes.

### Overexpression of *AfPXG* Increases the Size and Number of LDs

**FIGURE 3:**
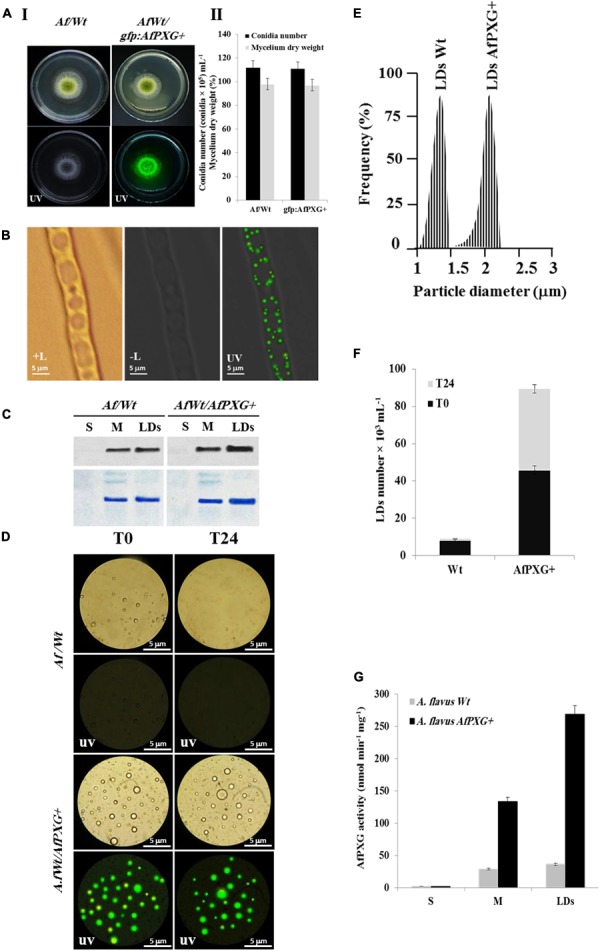
Overexpression of AfPXG is associated with increased numbers of lipid droplets (LDs) in *A. flavus*. **(A)** Growth of AfPXG-overexpressing lines compared with WT. The *AfPXG* gene was overexpressed under the control of *AdhI* promoter as described in Section “Materials and Methods.” *GFP* was used as a reporter gene to evaluate the efficiency of target gene overexpression. The *gfp:AfPXG+* line shows fluorescence under UV similar to the WT. **(B)** Light and fluorescent micrographs of isolated LDs from fungal tissue of *A. flavus* AfPXG+ line compared with WT. Purified LDs suspended in 100 mM potassium pyrophosphate at pH 7.4 were observed under a LEICA MPS60 microscope and images viewed at a magnification of 40× immediately after preparation (T0) or 1 day later (T24). Bar represents 5 mm. **(C)** Immunodetection of overexpressed-AfPXG in different subcellular fractions, supernatant (S), microsomes (M), and LDs compared with their respective fractions isolated from the WT. An anti-PXG antibody was used at dilution of 1:500 in TBS buffer (pH 7.4). Horseradish peroxidase-conjugated anti-mouse IgG diluted 1:2000, served as the secondary antibody. The signal was detected in a Pharos FX molecular imager (Bio-Rad). Loading control are gels stained with Coomassie Brilliant Blue. **(D–F)** The purity, size, and number of LDs were evaluated by a Flow Cytometer at T0. **(G)** Evaluation of PXG activity (hydroxylation of aniline) in the supernatant (S), microsomes (M) and LDs from *A. flavus* AfPXG+ line compared with their respective fractions isolated from the WT. All measurements were done in triplicates. Results are means ± SD (*n* = 3).

To gain more insights into the biological functions of *AfPXG* in fungal development and more particularly in the sporulation and AF biosynthesis, we used protoplasts of the line AfPXGΔ to stably overexpress the *AfPXG* gene fused with the GFP as a reporter gene, under the control of *AdhI* promoter. As shown in **Figure 3A**, the growth of AfPXGΔ was effectively reestablished when *AfPXG* gene expression was restored. This line, referred as *gfp::AfPXG+*, efficiently overexpressed the fusion protein AfPXG/GFP as shown under UV-light imaging (**Figure 3AI**) and colonies grew and sporulated similarly to WT lines (**Figure 3AII**). When mycelia of the *gfp::AfPXG+* strain were examined under fluorescent microscopy, LDs labeled with GFP were clearly localized in rings around the vacuoles, implying a close association between the two organelles (**Figure 3B**). Overexpression of AfPXG was immunodetected in microsomal (M) and in LD fractions isolated from the line *gfp::AfPXG+* at higher levels compared the respective subcellular fractions isolated from WT strains (**Figure 3C**). In contrast, no signal was detected in the supernatant (S) for both lines.

As AfPXG proteins were mainly targeted to LDs, we investigate the biological feedback of overexpression of AfPXG on the fungal LDs. Interestingly, compared with the WT, the line *gfp::AfPXG+* produced more LDs (detected as green-fluorescent spherical bodies) when examined under fluorescence microscopy immediately after isolation (T0) (**Figure 3D**). The LDs fractioned from the line *gfp::AfPXG+* were highly stable when examined 24 h (T24) after preparation. In contrast, LDs from the WT strain had completely disappeared after 24 h (**Figure 3D**). In terms of size and number, the LDs isolated from the line *gfp::AfPXG+* were about double the diameter and 5.5-fold more numerous compared with LDs of the WT strain at T0 (**Figures 3E,F**). Finally, the overexpression of AfPXG was followed by a significant increase in PXG enzymatic activity. As shown in **Figure 3G**, the microsomal (M) and the LD fractions from *AfPXG+* lines showed 4.5- and 7.4-fold, respectively, higher PXG activities compared with similar fractions isolated from the WT strain. Taken together, these data indicate that the overexpression of AfPXG results in the stable accumulation of more numerous and larger LDs with higher PXG activities than the control strain.

### Overexpression of *AfPXG* Increases the Biosynthesis and Secretion of AF

**FIGURE 4:**
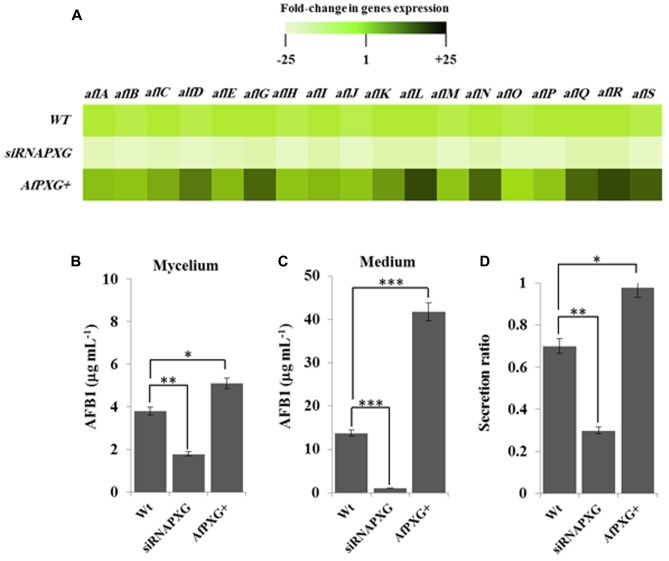
The increased lipid droplet (ILD)-phenotype in *A. flavus* AfPXG+ is positively correlated with biosynthesis and secretion of AF. **(A)** Transcripts levels of the AFB1-biosynthesis genes in fungal tissue of the *A. flavus* AfPXG+ line compared with *AfPXG2*-silenced and WT lines were evaluated by qRT-PCR. Three measurements were taken in three cDNAs prepared from three individual fungal growth for each line. Each point was triplicated and the average of *C*_T_ was taken. The relative quantification RQ=2^(-ΔΔC_T_^) of the target gene was calculated using installed software from an Applied Biosystems StepOne cycler, United States. The color scale (white–green–black) indicates relative changes of transcript abundance of -25-, 1-, and +25-fold, respectively. For each gene, the expression level in WT was defined as 1, and the corresponding abundance changes under treatments were calculated directly using the software installed in the Applied Biosystems qPCR system. **(B,C)** Quantification of AFB1 production in the fungal mycelium and in the culture medium of *A. flavus* AfPXG+ line compared with *AfPXG2*-silenced and WT lines. **(D)** Secretion ratio of AFB1 by the three fungal lines (calculated as ratio of AFB1 in the medium to AFB1 in medium plus mycelia in a 100 mL culture). All measurements were performed in triplicate and the presented data are means ± SD (*n* = 3). The differences in AF production between lines were significant when analyzed by *t*-test (^∗^*P* < 0.05; ^∗∗^*P* < 0.01; ^∗∗∗^*p* < 0.001).

We next evaluated the effect of AfPXG-overexpression on the aflatoxigenecity of *A. flavus* compared to WT as well as to the AfPXG-silenced strain, siRNAPXG. First, at the transcriptional level, several key genes in the AF biosynthesis pathway were significantly upregulated. Notably, *aflC* (*pksA*), *aflD* (*nor-1*), *aflG* (*avnA*), *aflK* (*vbs*), *aflL* (*verb*), *aflN* (*verA*), *aflQ* (*ordA*), and *aflR* (*apa-2*), which are crucial genes involved in AF biosynthesis, were highly expressed in the AfPXG-overexpressed line (*AfPXG+*) (**Figure 4A**). Transcripts of these genes were approximately 15- to 20-fold more abundant in *AfPXG+* tissues compared with the WT strain. Conversely, the AfPXG-silenced strain (siRNAPXG) showed a serious deficiency in the expression of these genes (**Figure 4A**).

To evaluate the biochemical feedback on transcriptional regulation for AF biosynthesis genes, we quantified the amount of AF in fungal mycelia, and in the medium of the two lines, *AfPXG+* and siRNAPXG, compared with the WT and hence determined the ratio of biosynthesis/export for AF in each of the lines. While mycelia of *AfPXG+* produced AFB1 (5.1 μg mL^-1^) more actively than WT mycelia (3.8 μg mL^-1^), mycelia of siRNAPXG produced much less (1.8 μg mL^-1^) (**Figure 4B**). The variation in the amount of AF exported into the medium became more evident between lines. **Figure 4C** shows that the line *AfPXG+* exported a high amount of AFB1 (about 41.8 μg mL^-1^) and the strain siRNAPXG exported only 1.1 μg mL^-1^ compared with the WT, which exported about 13.7 μg mL^-1^. From the previous data, we calculated the secretion ratio of AF for each line. Interestingly, we found that *AfPXG+* effectively secreted AF with a ratio of about 0.98 compared with the WT (0.71) but this ratio was only 0.3 in the strain siRNAPXG (**Figure 4D**). These results suggest that AfPXG is pivotal for both biosynthesis and extracellular export of AF.

### LDs Can Efficiently Sequester AF in *A. flavus*

**FIGURE 5:**
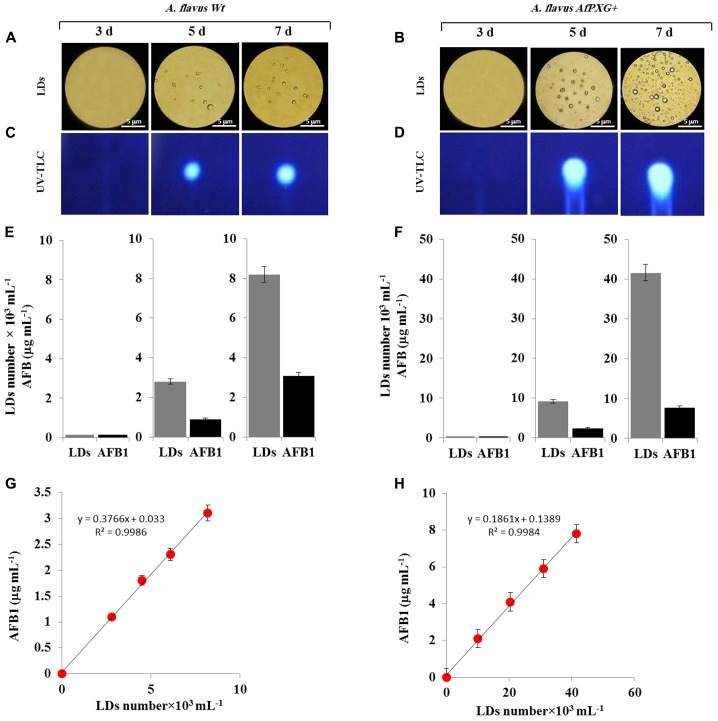
Purified and stable lipid droplets (LDs) isolated from the line AfPXG+ effectively sequester AF. **(A,B)** Light micrographs of isolated LDs from fungal tissue of *A. flavus* WT or *A. flavus* AfPXG+ line, respectively, on days 3, 5, and 7. Purified LDs suspended in 100 mM potassium pyrophosphate, pH 7.4 were observed under a LEICA MPS60 microscope and images were taken at a magnification of 40×. Bar represents 5 mm. **(C,D)** TLC-analysis of AFB1 in LD fractions isolated from WT and AfPXG+ lines. **(E,F)** Quantitative data for LD number and content of AFB1 in fractions isolated from WT and AfPXG+ lines. **(G,H)** Linear relationship between the number of LDs and their AFB1 content. AFB1 was extracted and analyzed by UV-HPLC as described in Section “Materials and Methods.” Measurements were done in triplicate. Values are the means ± SD (*n* = 3).

The AfPXG-overexpressing line of *A. flavus* had elevated numbers of LDs and an increased capacity for AF export. It is of interest to ascertain whether there is a biochemical connection between production of LDs and export of AF from fungal cells. One interesting possibility is that fungal LDs can sequester and/or export AF molecules in an analogous manner to that reported for other lipophilic molecules in algal and plant cells (Boucher et al., 2008; Bonsegna et al., 2011; Gwak et al., 2014; Chang et al., 2015; Shimada et al., 2015; Hanano et al., 2016). The first way to test this hypothesis was to obtain a fraction of LDs with high purity and stability. We then followed the kinetics of assembly and accumulation of LDs in fungal tissue of *AfPXG+* compared with WT. The micrographs taken for fresh preparations of LDs on days 3, 5, and 7 after inoculation, showed that both WT and *AfPXG+* accumulated the LDs with similar kinetics. However, the number of LDs was much higher in *AfPXG+* on days 5 and 7 (**Figures 5A,B**).

Next, pure LDs produced by WT and *AfPXG+* lines were used to extract any associated AF. This demonstrated that in both lines the LDs contained large amounts of AFB1 as shown qualitatively by TLC (**Figures 5C,D**). The intensity of the UV-fluorescent spots increased markedly on days 5 and 7 with higher amounts of fluorescence detected in extracts from LDs of *AfPXG+* compared to WT controls. In quantitative terms, the LD number was higher in the mycelia of *AfPXG+* compared with WT (9.2 versus 2.8 × 10^3^ mL^-1^) and (39.6 versus 8.2 × 10^3^ mL^-1^) on days 5 and 7, respectively. Likewise, the amount of AFB1 was higher in extracts from LDs from *AfPXG+* compared with WT (0.9 versus 2.4 μg mL^-1^) and (3.1 versus 7.8 μg mL^-1^) on days 5 and 7, respectively (**Figures 5E,F**). The relationship between LD number and the amount of bound AF showed a linear correlation with a high strength of association (*r*^2^ = 0.9982 versus *r*^2^ = 0.9968) for *AfPXG+* and WT, respectively (**Figures 5G,H**). These data suggest that pure LDs isolated from fungal tissues can effectively capture and sequester AF and that this ability is positively correlated with LD number.

### AfPXG Enzymatic Activity and Integration into LDs Are Both Essential for Biosynthesis and Secretion of AF

**FIGURE 6:**
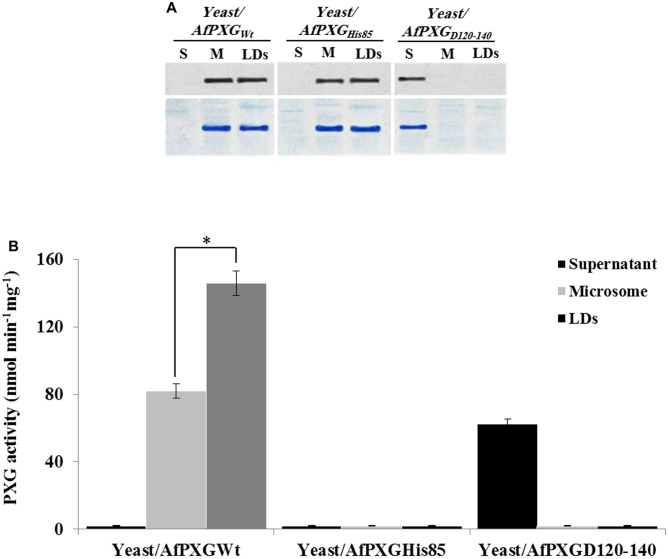
Expression, subcellular localization and enzymatic activity of AfPXG variants. **(A)** Immunodetection of the mutated proteins AfPXG_His85_ and AfPXG_D126-140_ or native AfPXG_WT_ in different subcellular fractions, supernatant (S), microsomes (M), and lipid droplets (LDs) isolated from the recombinant yeast. An antibody anti-PXG was used at dilution of 1:500. A horseradish peroxidase-conjugated anti mouse IgG diluted 1:2000, served as the secondary antibody. Loading control are gels stained with Coomassie Brilliant Blue. **(B)** Evaluation of the PXG activity (hydroxylation of aniline) in the respective subcellular fraction isolated from the recombinant yeast/AfPXG_His85_, yeast/AfPXG_D126-140_ and yeast/AfPXG_WT_. All measurements were done in triplicate. Values are the means ± SD (*n* = 3). The differences in PXG activity between microsomes and LDs were significant (^∗^*P* < 0.05).

The involvement of AfPXG in the regulation of AF biosynthesis and export can possibly be mediated via LDs, where both AF and AfPXG are targeted, and also through its enzymatic activity as this fungal caleosin exhibits a peroxygenase activity (Hanano et al., 2015). To test whether one or the both features affect the biosynthesis of AF and/or its export, we generated two mutants of AfPXG in yeast. One was mutated in Histidine residue 85 (His85) (AfPXG_His85_), a residue essential for heme binding and catalytic activity (Hanano et al., 2006). In the other mutant, the transmembrane and LD-binding domain located between Asparagine 126 and Aspartic acid 140 (AfPXG_D126-140_) was removed. Heterologously expressed in yeast, variants AfPXG_His85_ and AfPXG_WT_ were immunodetected in microsomal and LD fractions but were absent from the supernatant (**Figure 6A**). In contrast, the variant AfPXG_D126-140_ was only detected in the soluble fraction. Protein expression and enzymatic activity in the variants were confirmed by PXG assays. While microsomes and LDs from AfPXG_WT_ exhibited a typical PXG activity, no activity was found in the respective fractions from AfPXG_His85_ (**Figure 6B**). The PXG activity of the variant AfPXG_D126-140_ was found only in the supernatant and was slightly less than the activity of AfPXG_WT_ (**Figure 6B**).

**FIGURE 7:**
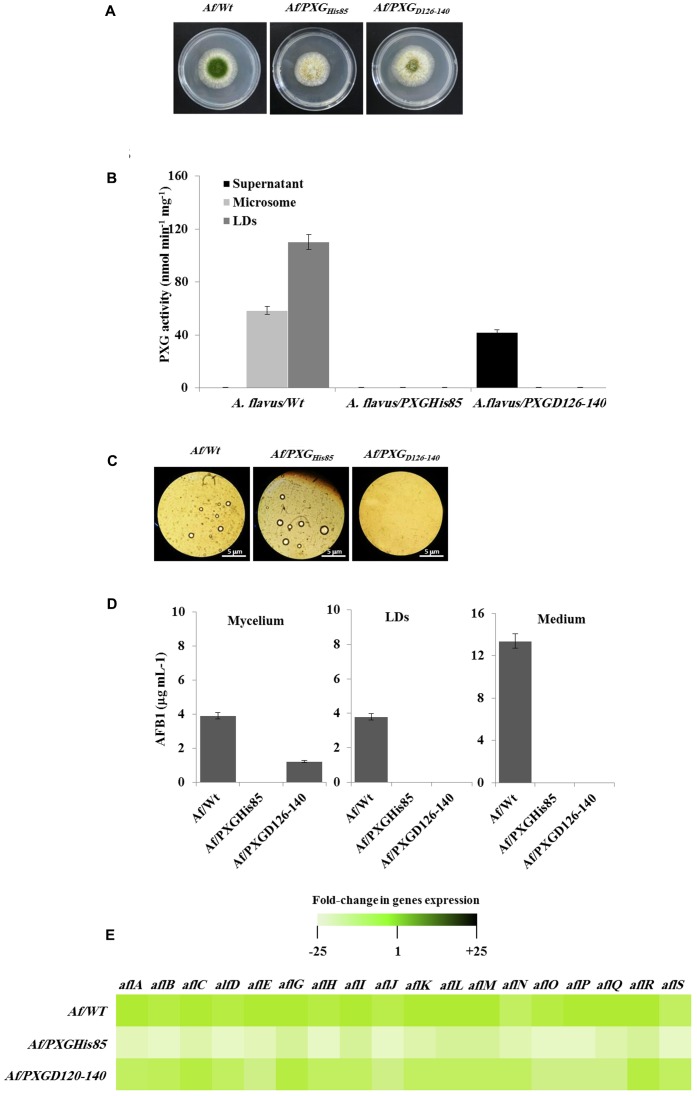
Effect of AfPXG enzymatic activity and the absence of its transmembrane domain on biosynthesis and/or export of AFB1. **(A)** Growth of *Af/AfPXG_His85_* We have changed ““textitAf/AfPXGHis85” and ““textitAf/PXGD126–140” as ““textitAf/AfPXG$_“textHis85′′and“∖textitAf/PXG$_∖textD126-140_”, respectively here and throughout. Kindly confirm if this if fine., *Af/PXG_D126-140_* lines compared with *Af/WT*. **(B)** PXG activity in supernatant (S), microsomes (M), and lipid droplets (LDs) isolated from the *Af/AfPXG_His85_*, *Af/PXG_D126-140_* lines compared with *Af/WT*. **(C)** Light micrographs of the isolated LDs from fungal tissue *Af/AfPXG_His85_*, *Af/PXG_D126-140_* lines compared with *Af/WT*, respectively, on day 7. Recovered LDs were observed under a light microscope and the images were taken at a magnification of 40×. Bar represents 5 mm. **(D)** Quantification of AFB1 in the fungal mycelium, LDs and in the culture medium of *Af/AfPXG_His85_*, *Af/PXG_D126-140_* lines compared with *Af/WT*, respectively, on day 7. **(E)** Transcript levels of AFB1-biosynthesis genes in fungal tissue of *Af/AfPXG_His85_*, *Af/PXG_D126-140_* lines compared with *Af/WT* were evaluated by qRT-PCR. The color scale (white–green–black) indicates relative changes of transcript abundance of –25-, 1-, and +25-fold, respectively. For each gene, the expression level in *Af/WT* was defined as 1. The measurements were done in triplicate. Values are the means ± SD (*n* = 3).

Having confirmed the expression and activities of AfPXG variants in yeast, we generated a stable line of *A. flavus* for each variant, Af/PXG_rmHis85_ and Af/PXG_D126-140_. As shown in **Figure 7A**, line Af/PXG_His85_ grew as fast as the control line Af/WT but was still unable to sporulate. In contrast, line Af/PXG_D126-140_ grew normally and formed spores, albeit fewer than in Af/WT. To evaluate the biochemical phenotype of both lines, we measured PXG activity in their respective supernatant, microsomal and LD fractions. No PXG activity was found in any of the subcellular fractions isolated from the line Af/PXG_His85_, whereas substantial PXG activity was only detected in the supernatant from line Af/PXG_D126-140_ (**Figure 7B**). This is unusual as PXG activity is normally only found in microsomal and LD fractions. Finally, the capacity of both lines to accumulate LDs was examined. **Figure 7C** shows that line Af/PXG_His85_ accumulates LDs similarly to line Af/WT, whereas line Af/PXG_D126-140_ failed to accumulate detectable LDs over 7 days of culture.

The accumulation of AF was followed in mycelia, LDs and in the culture medium of both lines compared with the WT. Interestingly, no AF was detected in mycelia, in LDs or in the culture medium from line Af/PXG_His85_. In contrast, about 1.2 μg mL^-1^ of AFB1 was found in mycelia of line Af/PXG_D126-140_ while no AFB1 was detected in either LDs or the external medium (**Figure 7D**). As expected, in control Af/WT cultures, AFB1 was found in mycelia, LDs and especially in the external medium. The failure of line Af/PXG_His85_ to synthesize AF was confirmed at the transcriptional level by examining the gene cluster involved in its biosynthetic pathway. The heat map in **Figure 7E** shows that line Af/PXG_His85_ had a net decrease (20- to 24-fold) in transcript levels for all target genes compared to lines Af/WT and Af/PXG_D126-140_, both of which had similar transcriptional profiles (**Figure 7E**). These results suggest that AfPXG regulates AF biosynthesis via its peroxygenase activity but that its integration within LDs is also essential for AF export from mycelial cells.

### Expression of a Plant OLEO1 in Fungi Enhances Secretion, But Not the Biosynthesis, of AFB1

**FIGURE 8:**
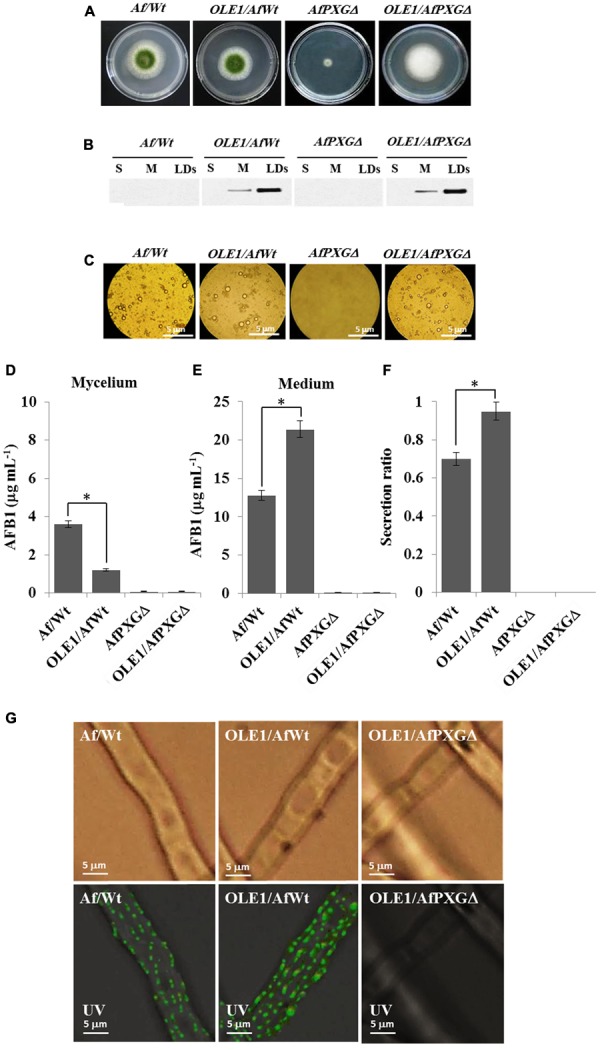
Effect of the oleosin1 (OLE1) expression on fungal growth and AFB1 secretion. **(A)** Five-day-old cultures of transformants OLE1/AfWt and OLE1/AfPXGΔ compared with their respective background lines AfWt and AfPXGΔ. **(B)** Immunodetection of OLE1 protein in different subcellular fractions, supernatant (S), microsomes (M), and lipid droplets (LDs) isolated from the transformants OLE1/AfWt and OLE1/AfPXGΔ compared with their respective background lines. **(C)** Light micrographs of the isolated LDs from the respective lines. **(D,E)** Quantification of AFB1 production in fungal mycelia and in the culture medium of transformants compared with background lines. **(F)** Secretion ratio of AFB1 by both transformants (calculated as ratio of AFB1 in the medium to AFB1 in medium plus mycelia in a 100 mL culture) compared with control lines. **(G)** Fluorescence image showing aflatoxin on the cell surface. The immunodetection of AFB1 was performed by its primary antibody coupled with a FITC-labeled secondary antibody. The measurements were done in triplicates. Values are means ± SD (*n* = 3). The differences in AF production between lines were significant when analyzed by *t*-test (^∗^*P* < 0.05).

To better define the role of LDs and their associated proteins in the secretion of AF, an *A. thaliana* oleosin (OLE1) (At4g25140, gene ID: 828617), which is a non-peroxygenase LD-associated protein, was expressed in two background lines of *A. flavus*, the wild-type (AfWt) and the mutant (AfPXGΔ). As shown in **Figure 8A**, there were no morphological differences between colonies from the control line and the line expressing OLE1 (OLE1/AfWt). However, there was a considerable increase in vegetative growth in mutant lines expressing OLE1 (OLE1/AfPXGΔ). In both lines, the expressed OLE1 was highly localized in the LD fraction, with a smaller amount present in the microsome (M) fraction and none in the soluble (supernatant) fraction (S) (**Figure 8B**). Expression of the exogenous plant oleosin was accompanied with a net increase in the accumulation of LDs in fungal cultures as shown in **Figure 8C**. Mycelia from OLE1/AfWt lines contained about threefold less AFB1 compared with the wild type AfWt strain but the OLE1/AfWt lines also secreted substantially more AFB1 in the medium (21.4 versus 12.8 μg mL^-1^) (**Figures 8D,E**). This was correlated with a higher secretion ratio of AFB1 in the line OLE1/AfWt (about 0.95) compared with AfWt (0.72) (**Figure 8F**). It was also noted that the caleosin-deficient line OLE1/AfPXGΔ was unable to produce any detectable AFB1, suggesting that while exogenous plant oleosin was able to stimulate LD accumulation it was unable to rescue the AF-deficient phenotype caused by the absence of AfPXG caleosin. Furthermore, while we detected more active immunofluorescence, resulting in the immunodetection of AFB1 by its primary antibody coupled with a FITC-labeled secondary antibody, on the mycelium surface of the OLE1/AfWt compared with AfWt, both AfPXGΔ and OLE1/AfPXGΔ did not show any detectable fluorescence (**Figure 8G**). These data strongly suggest that both fungal LDs and caleosins are intimately involved in the production and secretion of AF.

## Discussion

Caleosins are plant and fungal peroxygenases with highly conserved calcium-binding EF hand and lipid-binding domains, invariant heme-coordinating histidine residues and several putative phosphorylation sites (Naested et al., 2000; Hanano et al., 2006, 2015; Song et al., 2014). We have shown that caleosins from both plants and fungi bind to bilayer membranes via a transmembrane domain, and also to the phospholipid monolayer membrane surrounding intracellular LDs (Partridge and Murphy, 2009; Hanano et al., 2015). Within fungi, caleosins have been experimentally verified as both LD- and microsomal-associated proteins in a range of species including the ascomycetes, *A. flavus* (Hanano et al., 2015) and *B. bassiana* (Fan et al., 2015) and the basidiomycete, *Rhodosporidium toruloides* (Zhu et al., 2015). Expression of caleosin genes in response to biotic and non-biotic stresses has also reported in numerous fungal species including *Aspergillus oryzae* (Machida et al., 2005; Akao et al., 2007), *U. maydis* (Tollot et al., 2016), and *E. necator* (Wakefield et al., 2011). Caleosin gene expression is strongly upregulated during fungal infection (Fan et al., 2015; Hanano et al., 2015; Ortiz-Urquiza, 2016) suggesting that one of their roles in pathogenic fungi is to mediate interactions with the host. In plants infected by fungal pathogens, caleosin gene expression is also induced in the host plant (Partridge and Murphy, 2009; Sham et al., 2014; Hanano et al., 2015; Shimada et al., 2015). It appears, therefore, that plant and fungal caleosins might each act during the crosstalk within host–pathogen interactions.

Caleosin-like sequence(s) are present in most fungal genomic sequences, ranging from the most complex Dikarya (including all *Aspergillus* spp.) to the basal or derived fungal taxa, Cryptomycota and Microsporidia (**Figure 1**). These sequences include each of the four characteristic caleosin functional domains as outlined above (Murphy, 2012; Hanano et al., 2015, 2016). One of the few major fungal families where caleosin genes are absent from their genomes is the Saccharomycetaceae, which includes the intensively studied model species, *S. cerevisiae*. It is possible that in these particular yeasts the caleosin genes have been secondarily lost as part of a generalized metabolic streamlining that occurred when they evolved from a multicellular to a unicellular organization (Ratcliff et al., 2012). However, the genomes of some closely related budding yeasts, such as *Lipomyces starkeyi* (Saccharomycetales) do contain caleosin-like genes. Moreover, even in caleosin-deficient yeasts such as *S. cerevisiae*, the ectopic expression of caleosin genes from other species, including plants and fungi (see **Figure 6**), results in the faithful biosynthesis and processing of the exogenous protein, its targeting to LDs/microsomes and an authentic peroxygenase activity in the yeast cells (Froissard et al., 2009; Jamme et al., 2013; Hanano et al., 2015).

In the present study, we demonstrate that the *A. flavus* caleosin is involved in several key developmental and environmental responsiveness processes and its deletion led to a 92% reduction in mycelial dry weight and complete abolition of both AF production and spore formation (Supplementary Figure S1). Since AF production is known to occur in response to oxidative stress (Jayashree and Subramanyam, 2000; Reverberi et al., 2012; Fountain et al., 2014; Luo et al., 2016), we attempted to rescue the AF-deficient phenotype by chemically inducing oxidative stress in caleosin-deleted strains (**Figure 2**). The failure to restore AF production by applying external oxidative stress to these strains indicates that the presence of a functional caleosin gene is required for AF biosynthesis and/or secretion. Since caleosin-mediated upregulation of several AF biosynthesis-cluster genes could not be rescued by exogenous oxidative stress, it is possible that one function of caleosins is to generate a highly specific suite of oxylipins that serve to initiate the transcription of this subset of AF biosynthesis-cluster genes. These conclusions are reinforced by overexpression of the *A. flavus* caleosin gene in the null strain, AfPXGΔ, which led to increased mycelial dry weight and expression of several AF biosynthesis-cluster genes plus a >3-fold increase in AF secretion into the medium (**Figure 4**) and demonstrates the importance of caleosins for the regulation of overall fungal development and AF metabolism in *A. flavus*. We also demonstrate that stimulation of LD accumulation alone in caleosin-deficient fungal cells is not sufficient to restore AF biosynthesis and secretion (**Figure 8**), so that both caleosin and LDs are required for optimal AF metabolism in *A. flavus*.

Cytosolic LDs are increasingly recognized as dynamic multifunctional organelles with multiple roles in organisms including bacteria, archaea plus all of the major eukaryotic clades (Murphy, 2012; Radulovic et al., 2013; Gao and Goodman, 2015). In addition to its stimulation of AF metabolism, overexpression of the *A. flavus* caleosin gene resulted in a dramatic increase in the number and size of LDs in fungal cells (**Figures 3A–D**). There was also a huge increase (>7-fold) in peroxygenase activity compared to WT colonies with about 12% of the activity associated with microsomes and 88% with LDs (**Figures 3E,F**). Expression in yeast of recombinant *A. flavus* caleosin variants containing mutagenized versions of the heme-coordinating histidine and lipid-binding domains, respectively abolished or severely reduced peroxygenase activity and LD or microsomal membrane binding compared to controls (**Figure 6**). This indicates that both lipid binding and heme coordination are important for caleosin function in such fungi.

The role of LDs as transient stores of lipophilic and non-lipophilic compounds, from small metabolites to large proteins, is well established (Murphy, 2012; Chang et al., 2015; Gao and Goodman, 2015; Kory et al., 2016). In some cases, such as in the essential oil glands of some plants, this involves the trafficking and secretion of complex mixtures of a range of compounds, such as terpenoids and fatty acid derivatives, which are initially sequestered in LDs. In some cases, these LDs move inside small vesicles that are secreted via exocytosis (Gersbach, 2002; Rehman et al., 2016). In the fungus, *U. maydis*, there is evidence of a Rab-mediated fusion/binding mechanism between LDs and early endosomes (Meadows, 2012) and of communication between the contents of the two compartments (Goodman, 2008). Further evidence for this comes from reports that both peroxisomes and LDs can “hitch-hike” on early endosomes moving vectorially along microtubules via motor kinesin-3 and dynein proteins (Guimaraes et al., 2015; Steinberg, 2016). There are also reports that plant LDs sequestering secretory metabolites, such as flavones and terpenes, are trafficked from their site of synthesis on the ER to the plasma membrane via a vesicular endomembrane system (Tanchak and Fowke, 1987; Tse et al., 2004; Guo et al., 2013). This is consistent with our observations here that LDs in *A. flavus* are effective at sequestering AF and in mediating their trafficking and eventual secretion, most likely in conjunction with previously reported endosome components such as aflatoxisomes (**Figure 5**).

There are numerous reports of LD involvement in trafficking and secretion of products via the endomembrane system in other organisms. For example, in mammalian (HeLa) cells infected with the obligate intracellular bacterial pathogen, *Chlamydia trachomatis*, cytoplasmic LDs are translocated out of the host cell and into the lumen of the parasitophorous vacuole of the bacterium (Cocchiaro et al., 2008). In mammalian adipocytes, the formation, trafficking, and secretion of cytosolic and ER-lumen located LDs involves a cascade of proteins acting on the *trans*-Golgi endomembrane system and there are numerous report of LDs carrying proteins relation to vesicle trafficking including secretion (Tsitsigiannis et al., 2004; Natter et al., 2005; Bouchez et al., 2015; Kory et al., 2016; Yoneda et al., 2016; Meyers et al., 2017). These and other studies from various biological systems demonstrate that LDs, possibly carrying specific cargoes, can be taken up into intracellular vacuoles and can also be secreted via exocytosis. In one of the original investigations into AF secretion in *A. parasiticus* where the roles of aflatoxisomes was proposed, it is interesting that these secretory vesicles were surrounded by small osmiophilic droplets with a similar morphology to LDs (Chanda et al., 2009). In line with these observations, our results showed a very similar localization of GFP-labeled LDs around or adjacent to the vacuolar membrane. Interestingly, no LDs were detected inside the vacuoles, which suggests that AF might be trafficked between vacuoles and LDs rather than entire AF-laden LDs being engulfed by vacuoles.

In terms of the interactions between LDs and vesicles it is interesting that biophysical model studies have shown that lipids such as phosphatidylcholine can form several phases in aqueous solution, including as LDs, micelles, and/or vesicles (Wang and He, 2009). This suggests at least a theoretical possibility that LDs could merge with vesicles as well as becoming engulfed by them. In addition to these biophysical considerations, there are well-established links between LDs and vacuoles/endosomes via their common associations with Rab proteins and other protein components of vesicle trafficking pathways in several organisms, including fungi (see Gao and Goodman, 2015). Very recently it has even been shown that some specialized LDs are located with the nucleus of several mammalian cell types where they may be involved in sequestration of misfolded or unfolded hydrophobic proteins and/or as detoxification sites for hydrophobic compounds (Farese and Walther, 2016).

Our results demonstrate that in *A. flavus*, AF is at least partially sequestered in LDs that also contain caleosin, which is a peroxygenase. The fact that 90% of the AF in such fungal cultures is secreted into the external medium begs the question of whether the AF was still bound to LDs after it was secreted. As detailed above, there are numerous reports of the secretion of intact LDs from other biological systems. Moreover, it has been established that fungi are able to secrete a range of heme-thiolate peroxidases similar to caleosins (Hofrichter et al., 2010; Peter et al., 2011; Hofrichter and Ullrich, 2014). Since the *A. flavus* caleosin sequence does not contain any canonical target motifs for secretion, but does contain a lipid-binding motif, it seems likely that the protein is secreted as part of an LD cargo rather than via a classical endosome pathway. The uptake of potentially toxic metabolites or xenobiotic agents by LDs is increasingly recognized as an important aspect of cellular homeostasis. For example, Chang et al. (2015) have shown that the sequestration of toxins (in this case phototoxic perylenequinones) within LDs of the endolichenic fungus, *Phaeosphaeria* sp. [from the lichen *Heterodermia obscurata* (Nyl.) Trevis] was essential for fungal resistance to toxin-induced ROS. This study also showed that yeast (*S. cerevisiae*) cells were able to sequester the related fungicide, hypocrellin A, within their cytosolic LDs and thereby avoid being killed.

## Conclusion

**FIGURE 9:**
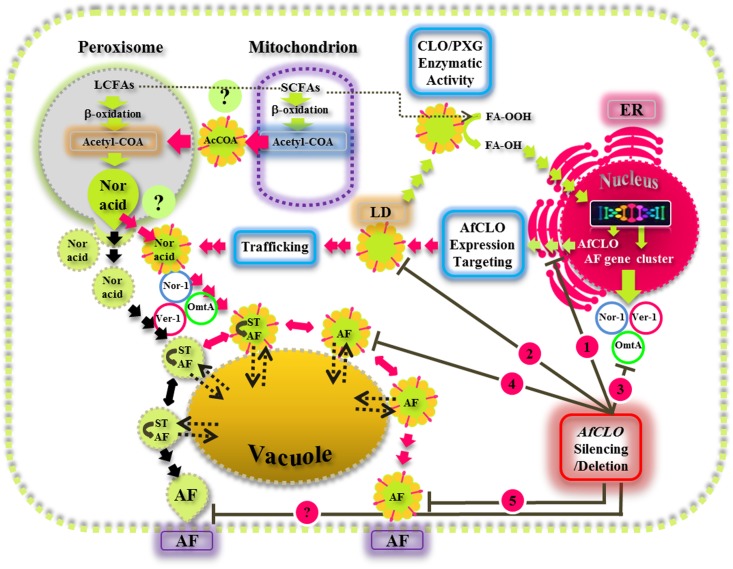
A model for the biosynthesis, intracellular trafficking, and secretion of aflatoxins via a concerted mechanism involving peroxisomes, lipid droplets (LDs), and endomembrane components. In this scheme, the caleosin/peroxygenase (CLO/PXG) enzyme, located on LDs, is a component of oxylipin metabolism that converts fatty acyl peroxides (FA-OOH) to fatty acyl hydroxides (FA-OH). This is part of the oxidative signaling cascade that regulates the expression of several components of the aflatoxin (AF) gene cluster located in the nucleus. AF biosynthesis begins in peroxisomes with the β-oxidation of long chain fatty acids (LCFAs) to acetyl-CoA which is then converted to the polyketide, Norsolorinic acid (Nor Acid), via polyketide synthase. Vesicles and LDs, loaded with Nor Acid, then bud off from peroxisomes (and possibly also from mitochondria) and are trafficked via CVT (cytoplasmic vacuole targeting) vesicles that contain middle and late aflatoxin enzymes such as Nor-1, Ver-1, and Omt-A. The aflatoxin-loaded vesicles fuse with secretory vesicles to form aflatoxisomes that can either be recycled via the vacuole or secreted from the cell (black arrows). In the alternative pathway that has been elucidated in this study, LDs can also become loaded with AF and can then either be recycled via the vacuole or secreted from the cell (red arrows). Silencing of the *AfCLO* gene results in the downregulation of several key AF gene cluster components, as well as much reduced LD accumulation and an almost total cessation of AF biosynthesis and secretion.

This study provides new insights on the biological functions of fungal LDs and their associated caleosin/peroxygenase in the regulation, biosynthesis, and export of AF in *A. flavus*. Our findings suggest that the biosynthesis, intracellular trafficking, and secretion of AF occurs via a concerted mechanism involving peroxisomes, LDs, and endomembrane components and this is summarized by the schematic model presented in **Figure 9**. This proposed mechanism involves the involvement of both the conventional aflatoxisome route (black arrows) and an alternative LD-mediated route for AF trafficking from peroxisomes to vacuoles and subsequent secretion (red arrows). In the latter pathway, LDs become loaded with AF and can then either be recycled via the vacuole or secreted from the cell. Silencing of the *AfCLO* gene results in the downregulation of several key AF gene cluster components, as well as much reduced LD accumulation and an almost total cessation of AF biosynthesis and secretion. We suggest that the LD/caleosin mechanism is essential to AF biosynthesis and secretion and that LD-associated AF trafficking provides an alternative pathway to the established aflatoxisome route.

## Author Contributions

AH led the work, designed all experiments in biochemistry and molecular biology, and co-wrote the manuscript. MA, IA, and MS carried out all the experimental work. FR and MH performed the bioinformatics analysis. DM designed the computational analysis and co-wrote the manuscript. All authors read and approved the final manuscript.

## Conflict of Interest Statement

The authors declare that the research was conducted in the absence of any commercial or financial relationships that could be construed as a potential conflict of interest.

## Abbreviations

AFB1: aflatoxin B1
AfPXG: *Aspergillus flavus* peroxygenase
CLO: caleosin
Gfp: green fluorescent protein
LDs: lipid droplets
LOX: lipoxygenase
NoxA: NADPH oxidase A
OLEO: oleosin
Ppo: psi factor-producing oxygenase
PXG: peroxygenase
ROS: reactive oxygen species
TAG: triacylglycerol.
