# Supplementary material for: The Peroxygenase Activity of the Aspergillus flavus Caleosin, AfPXG, Modulates the Biosynthesis of Aflatoxins and Their Trafficking and Extracellular Secretion via Lipid Droplets
Source: Front Microbiol. 2018 Feb 6;9:158. doi: 10.3389/fmicb.2018.00158 (PMC5808235; doi:10.3389/fmicb.2018.00158)
Supplement: Supplementary file 1 [file Data_Sheet_1.PDF]

Supplementary materials and methods

Bioinformatics Analysis/Gene characterisation

For sequence retrieval and identification of caleosin in fungal species, putative CLO sequences of *Aspergillus flavus* (AflCLO), *Erysiphe necator* (EnCLO), *Neurospora crassa* (NcCLO), *Magnaporthe oryzae* (MoCLO), *Beauveria bassiana* (BbCLO), *Ustilago maydis* (UmCLO), *Rhodotorula toruloides* (RtCLO), *Gonapodya prolifera* (GprCLO), *Rhizophagus irregularis* (RiCLO), *Allomyces macrogymus* (AmaCLO), *Rozella allomycis* (RaCLO) were obtained from NCBI (http://www.ncbi.nlm.nih.gov/) via local BLAST+ searches (Chen *et al.*, 2015). After scanning the sequences using InterProScan program and visually inspecting for the presence of CLO-like domains, one representative caleosin sequence from each species was selected for further analysis (http://www.ebi.ac.uk/interpro) (see Supplementary Table S4 for accession numbers and physical properties). Chemical and physical properties of caleosin proteins (Molecular Weight (MW), isoelectric point (pI), amino acid length) were computed using ExPASY (http://web.expasy.org/compute_pi/ (Gasteiger *et al.*, 2005). Multiple sequence alignments were performed using COBALT software version 2.1.0 (ftp://ftp.ncbi.nlm.nih.gov/pub/cobalt/) and ClustalOmega software version 1.2.2 (http://www.ebi.ac.uk/Tools/msa/clustalo/) (Sievers *et al.*, 2011). The multiple sequence alignments were inspected using CLC sequence viewer (http://www.clcbio.com) and are presented using RasMol colour codes (Sayle and Milner-White, 1995) in Figure 1C. The multiple sequence alignment of all the caleosin sequences revealed that the Ca^{2+} binding EF hand and lipid-binding domains were highly conserved in all fungal species. For motif, Intron/exon and phylogeny analysis, see supplementary information.

Motif, Intron/exon and phylogeny analysis

The identified caleosin sequences were analysed to discover motifs using expectation maximization technique (Bailey and Elkan, 1994). MEME software version 4.11.2 was used to investigate Ca^{2+} binding, lipid-binding domain and histidine motifs in caleosin sequences. Five possible motifs were searched using a width between 15-50 (Figure1A, 1B) (Bailey and Elkan, 1994). The Scipio program version 1.4 was used to identify gene structure of each caleosin sequence (Keller *et al.*, 2008) (Figure 1D). Intron-exon patterns were inspected using Webscipio and are presented in (Figure 1D). The caleosin sequences were utilised to construct a phylogenetic tree using ClustalW2 version 2.1 and COBALT. The tree was generated following a Bayesian Inference (BI), neighbour joining (NJ), and Unweighted Pair Group Method with Arithmetic Mean (UPGMA) methods. The tree topologies constructed using the three methods showed complete consistency. The NJ tree constructed using the ClustalW2 program was inspected using FigTree and shown in Figure 1E (Rambaut and Drummond, 2009).

Replacement and siRNA-silencing of *AfPXG* gene

The replacement of the *AfPXG* gene by the hygromycin-resistance gene (Hyg^r) in the genome of *A. flavus* NRRL3357 was performed by fusion PCR as recently described (Hanano *et al.*, 2015). Primers used for the PCR amplifications are listed in Table S3. The Hyg^r gene was amplified by PCR using as a template the binary vector pCAMBIA1381 (http://www.cambia.org/daisy/cambia/585.html) (NCBI accession number AF234302). The three PCR-products, *5'-UTR*, Hyg^r and *3'-UTR* were mixed and used as a template in a fusion PCR (Kuwayama *et al.*, 2002) (Fig. S2 A supplementary info). The fusion PCR product was analysed on agarose gels (1 %) then delivered into protoplasts of *A. flavus* strain NRRL3357 (wild type) by electroporation as described previously (Hanano *et al.*, 2015). Verification of the replacement of *AfPXG* with Hyg^r was done using Southern hybridization. The probe consisted

of *AfPXG* flanking sequences surrounding *Hyg*^{r} as described previously (Hanano *et al.*, 2015). In wild-type DNA cut with NcoI (lane WT), the probe hybridizes to bands of 16, 11.5 and 4.5 kb at the *AfPXG* locus as shown in S2A (see bottom panel). Lane PXGΔ shows DNA from a AfPXGΔ digested by NcoI whereas a single 16-kb fragment was detected (S2A, bottom panel). The positions of DNA size standards (kb) are at the left. The absence of *AfPXG* transcripts and the peroxygenase activity in the strain AfPXGΔ as well as the subsequent effects of AfPXG deletion on the fungal growth were confirmed (Fig. S1E). The siRNA primers, siRNAPXGF and siRNAPXGR (Table S3), used in this study were designed as described previously (Hanano *et al.*, 2015). Protoplasts were prepared from fungal conidia as described by Cheng and Belanger (Cheng and Belanger, 2000). Briefly, about 1×10^8 conidia of 7-day old cultures of *A. flavus* NRRL3357 were cultured onto PDA plates, harvested, washed with sterile water then suspended in 20 mL of a solution of 25 mM L-mercaptoethanol and 5 mM Na_2EDTA pH 8.0 under gentle shaking for 20 min at room temperature. The spores were collected by centrifugation for 5 min at 5000 rpm and pellets resuspended and incubated for 2 h at 25° C in 5 mL of digestion buffer (10 mM Na_2HPO_4, 1.2 M $MgSO_4$, 10 mg mL^{-1} Lysozyme) (Sigma-Aldrich). The protoplasts were filtered through a small mass of cotton (about 2 cm^3) then centrifuged at 5000 rpm for 15 min at 4° C. Cells were washed twice with 5 mL STC buffer (1M Sorbitol, 50 mM CaCl2, 50 mM Tris-HCl, pH 8), taken up in 1 mL of STC buffer, and divided into 50 μL aliquots.

Overexpression of *AfPXG* or OLE1

Overexpression of *AfPXG* was carried out under the control of *AdhI* promoter using *Gfp* as a reporter gene. The construct *AdhI/AfPXG/Gfp* was introduced into the genome of the AfPXGΔ. To that end, the *AdhI* promoter region was amplified using genomic DNA of *A. flavus* and

primers P7 and P8. In parallel, the full-length *Gfp* gene, encoding enhanced green fluorescent protein (GFP), was amplified by PCR using vector pEGFP-N1 (Clontech Laboratories) (http://www.addgene.org/vector-database/2491/) (NCBI accession number U55762) as a template with primers P9 and P10. The full length *AfPXG* gene was amplified using the recombinant yeast expression vector pVT102U (Vernet *et al.*, 1987) and primers P11 and P12. The three PCR-products, corresponding to *AdhI, AfPXG* and *Gfp*, were mixed and used as templates for fusion PCR (Kuwayama *et al.*, 2002) (Fig. S2A, supplementary data). The fusion PCR product (*AdhI/AfPXG/Gfp*) was analysed on an agarose gel (1 %), purified then used to replace the Hyg^r gene in the strain AfPXGΔ. The replacement was performed by fusion PCR approach as described above using P13, P14, P15 and P16 primers (Fig. S1 B, supplementary data). This approach allowed the function of AfPXG to be regained with enhanced expression under the control of *AdhI* promoter. The final fusion PCR product 5'-UTR*/AdhI/AfPXG/Gfp/*3'-UTR was delivered into protoplasts of the strain AfPXGΔ by electroporation as described previously (Hanano *et al.*, 2015). The resulting phenotype was characterized by PCR and sequencing of the insert using the primers P14 and P15, by observation of GFP fluorescence under a 365 nm UV light, and by measuring PXG activity and *Hyg* susceptibility. For OLE1 expression, the Oleosin 1 gene (OLE1) from *Arabidopsis thaliana* (AT4G25140, gene ID: 828617) was amplified by PCR from cDNAs of one-week old Arabidopsis seedlings. The fusion PCR product (*AdhI/OLE1*) was analysed on agarose gels (1 %), purified and sequence-verified. It was then introduced via electroporation into *A. flavus* protoplasts prepared from background lines AfWT and AfPXGΔ as described above using P13, P14, P16 and P17 primers (Fig. S2 C, supplementary data). After five days of culture, the transformants, referred to as OLE/AfWT or OLE1/ AfPXGΔ were subjected to analysis in respect of their morphology,

immunology, LD accumulation and aflatoxin production. All experiments were performed using three biological replicates.

Preparation of *A. flavus* subcellular fractions and Peroxygenase activities assay

Isolation of microsomal and LD fractions from fungal cells was performed essentially as described by Ferreira de Oliveira and coworkers (Record *et al.*, 1998;Ferreira de Oliveira *et al.*, 2010) with brief modification as described previously (Hanano *et al.*, 2015). In brief, five grams of fungal mycelium were ground into a brass mortar in the presence of liquid nitrogen until a fine powder was obtained. After the liquid nitrogen was evaporated, the total powder was immediately hydrated with 10 mL of buffer A (100 mM potassium pyrophosphate, 0.1 M sucrose and pH 7.4). The mixture was then gently homogenized for 5 min using an ultra-dispenser (T25 digital ULTRA-TURRAX, IKA laboratory, Germany) and centrifuged for 10 min at 10,000 × *g*, and the supernatant centrifuged at 100,000 × *g* for 1 h to produce a floating white pad layer, consisting of LDs, and a pellet, corresponding to microsomes. LDs were collected from the top of the tube using a Pasteur pipette and then carefully washed twice with 5 mL of buffer B (buffer A without sucrose). After a final centrifugation (100,000× *g* for 1 h), the LD fraction was suspended in 1 mL of buffer B and stored at 4 °C for further analysis. In parallel, microsomes were taken, homogenized, washed twice and finally suspended in 1 mL buffer B. Peroxygenase activity was assayed by oxygenation of aniline or thiobezamide substrates (Blee and Durst, 1987) and by epoxidation of [1-^{14}C] oleic and linoleic acids according to Blée and Schuber (Blee and Schuber, 1990).

Genes, primers and transcripts analysis

Table S1 summarizes the gene order, NCBI-accession number, gene name, enzyme name and the respective catalytic activity for the *Aspergillus flavus* NRRL3357 aflatoxins-biosynthesis

cluster genes. Nucleotide sequences of primers used in this section are listed in Table S2. For the expression studies of *AfPXG* genes, *A. flavus* was grown as described previously. Total fungal biomass was collected for total RNAs isolation using an RNeasy kit according to the manufacturer's instructions (Qiagen, Germany). DNA traces were removed by 2 units of RNase-free RQI DNase (Promega, USA) for 1 h at 37º C. RNAs were diluted to 50 ng μL^{-1} using RNase-free water and stored at - 80º C. cDNA synthesis was performed using M-MLV RT (Invitrogen) as described previously (Hanano *et al.*, 2015). Real-time PCR was carried out in 48-well plates using a StepOne cycler from Applied Biosystems, USA. The 25-µl reaction mixtures contained 0.5 µM of each target and reference genes primers, 12.5 µl of SYBR Green qRT-PCR mix (Bio-Rad, USA) and 2.5 µl of 10-fold diluted cDNA. qRT-PCR conditions were as previously described (Hanano *et al.*, 2015). Each point was triplicated and the average of C_T was taken. The relative quantification RQ $= 2^{(-\Delta\Delta CT)}$ of the target gene was calculated using installed software from an Applied Biosystems StepOne cycler, USA.

Analysis of LDs

Microscopic imaging was performed at a magnification of 40× under a LEICA MPS60 microscope using an Olympus FE-4000 camera. The purity of LD preparation, their native encapsulation and their number per mL were evaluated by a Flow cytometer (BD FACSCALIBUR, Biosciences, USA). LD size distributions (% frequency) were determined using a laser granulometer (Malvern Mastersizer S; Malvern Instruments, England) fitted with a 320 mm lens as described previously (White *et al.*, 2006).

Biomass and conidia number measurements

Fungal biomass was determined as described previously (Hanano *et al.*, 2015). Mycelium dry weights were subsequently evaluated according to Rasooli and Razzaghi-Abyaneh (Rasooli and Razzaghi-Abyaneh, 2004). Fungal growth inhibition (%) was calculated according to the following formula: Fungal growth inhibition (%) = [(Total control weight - Total sample weight)/Total control weight] ×100. In parallel, the total conidia fraction for each plate was harvested and taken up in 5 ml solution of 0.01 % Tween 80, diluted to 1:10, and counted using a hemocytometer.

SDS-PAGE and Western blotting

LD-associated proteins were isolated according to Katavic et al. (Katavic *et al.*, 2006) and analysed by SDS–PAGE using 12% polyacrylamide gels and electroblotted onto a PVDF membrane (Millipore) in a Semi-Dry Transfer Cell (Bio-Rad). Caleosins were immunodetected by incubating the membrane with a polyclonal antibody prepared from the complete sequence of the CLO1 caleosin isoform from *Arabidopsis thaliana*, as described previously (Hanano *et al.*, 2006;Hanano *et al.*, 2016). The signal was detected in a Pharos FX molecular imager (Bio-Rad).

Extraction, clean-up, TLC and HPLC analysis of aflatoxin

The extraction of aflatoxin (AF) was carried out according to Bertuzzi et al., (Bertuzzi *et al.*, 2011) using 100 mL of chloroform for one hour on a rotary-shaker and extracts were purified as described previously (Shannon *et al.*, 1983). Extracted AF samples were spotted onto a C_{18} reversed-phase TLC plate (Aluminium sheets 20 × 20 cm, 200 μm layer, Merck, Germany) and the chromatogram was developed using a solvent system of chloroform/acetone (90:10, v/v). After development, the spot with the same *Rf* value as the AFB1 standard was scraped off, re-extracted with chloroform, and evaporated to dryness under nitrogen. The extract was taken up

in 100 μL acetonitrile in an amber-coloured vial under refrigeration. AF was analysed using a Jasco LC-2000 plus series HPLC system (Jasco, USA) with a fluorescence detector (RF-10Axl, Shimadzu) (λexc 247 nm; λem 480 nm) and a C18 column (Eclipse XDB-C18 150 × 4.6 mm, 5 μm; Agilent, USA, column temperature 35° C) as described previously (Hanano *et al.*, 2015).

Supplementary data

Generation and characterization of *AfPXG*-deficient lines of *A. flavus*

Deletion of the *AfPXG* gene in *A. flavus* reference strain NRR3357 (ATCC Number: 200026) using the replacement *AfPXG :: Hyg*^{r} dramatically affects fungal growth and development (Fig S1A and B). Growth of this line, referred to as AfPXGΔ, was dramatically decreased and its dry weight did not exceed 8% of the wild-type strain and AfPXGΔ cultures failed to produce any visible spores after 7 days of culture. We then partially silenced *AfPXG* via a small interference RNA (siRNA) approach to obtain putative intermediate phenotypes. The *AfPXG*-silenced strain, siRNAfPXG, retained only 16 % of *AfPXG* transcript compared to control (Fig. S1C), showed a reduced growth (32% compared to control strain siRNACt), but was still unable to sporulate (Fig. S1B). Deletion or silencing of *AfPXG* was confirmed by measuring peroxygenase (PXG) activities in term of sulfoxidation, hydroxylation and epoxidation reactions. PXG activity was completely abolished in crude extracts of AfPXGΔ, and did not exceed 14% in crude extracts of siRNAfPXG compared to the control, siRNACt (Fig. S1D). AfPXG-deficient lines also had dramatically reduced aflatoxigenecity. While line siRNAfPXG produced only 21% of aflatoxin B1 (AFB1) compared with the control siRNACt, line AfPXGΔ failed to secrete any detectable AFB1 (Fig. S1E). Together, these data confirm and extend our previous results suggesting a pivotal role of AfPXG in the development and aflatoxigenecity of *A. flavus*.

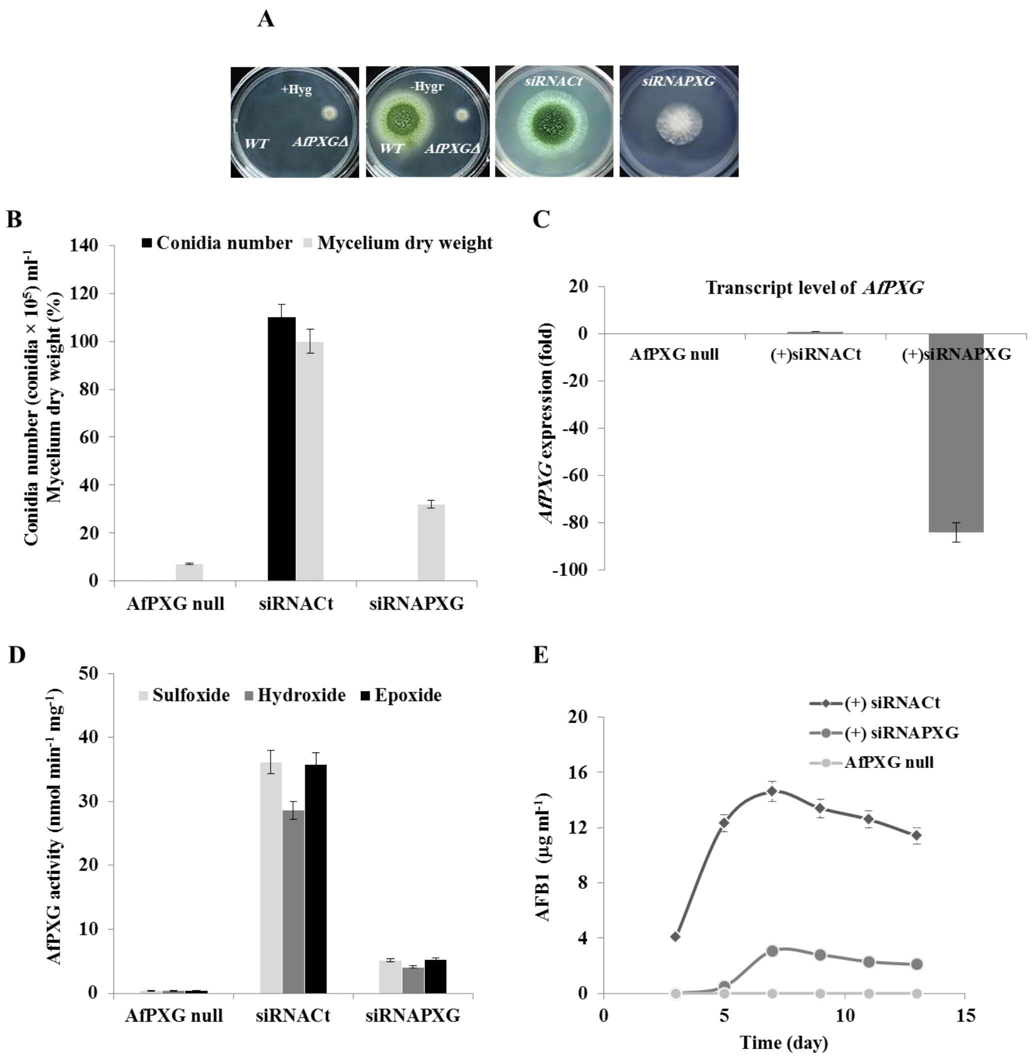

Figure S1. Phenotypic characteristics of *Aspergillus flavus* NRRL3357 following silencing or deletion of the *AfPXG* gene

(A) Morphology of fungal phenotypes grown on YPD plates for 4 days at 28° C. **(B)** Evaluation of conidia number (black columns) and dry weight (light-grey columns) in *AfPXG*-deficient and control strains. **(C)** *AfPXG* transcripts were quantified by qRT-PCR. **(D)** Peroxygenase (PXG) enzymatic activity in the total crude extract of fungal growth. Sulfoxidation of thiobenzamide (light-gray columns), hydroxylation of aniline (dark-gray columns) and epoxidation of [1-^{14}C]

oleic acid (black columns). Results are the means ± S.D. (n = 3). **(E)** Time course of AFB1 production in *AfPXG*-deficient lines compared with WT. AFB1 was extracted and analyzed on days 2, 5, 7, 9, 11 and 13 after inoculation as described in Methods. Measurements were done in triplicate. Values are means ± S.D. (n = 3). The significance of the differences between WT and other lines were analyzed by t-test ($^{*}P < 0.05$; $^{**}P < 0.01$).

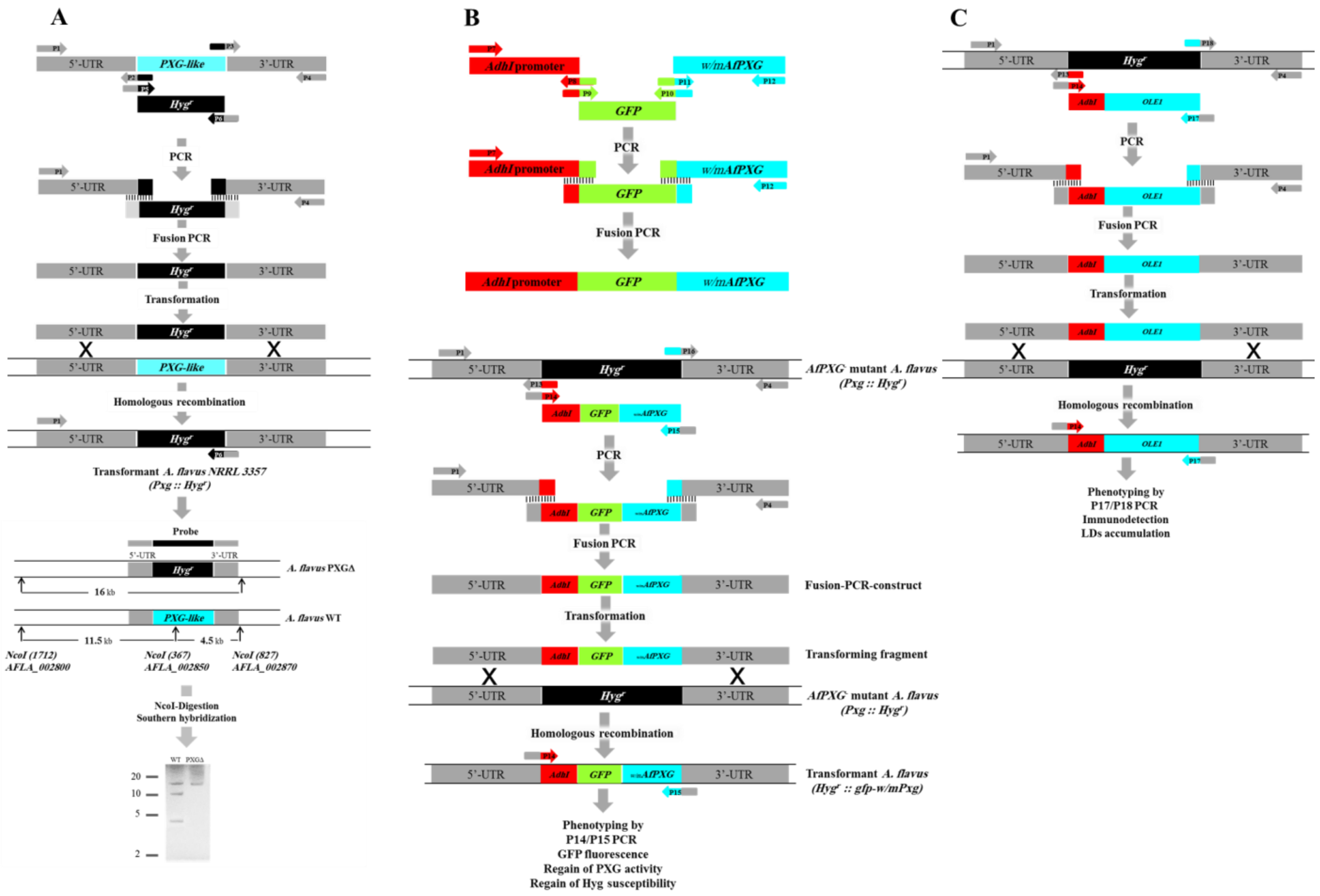

Figure S2: Strategy for replacing *PXG* by Hyg^{r} **(A)**, overexpression of PXG **(B)** and overexpression of OLE1 **(C)** by Fusion PCR-construct.

Supplementary Tables

Table S1 *Aspergillus flavus* NRRL3357 AF-biosynthesis cluster genes.

Gene order	Accession N.[a]	Gene name	Enzyme name	Catalytic activity
aflA	XM_002379907	*fas-2*	Fatty acid synthase α-subunit	Acetate › polyketide
aflB	XM_002379906	*fas-1*	Fatty acid synthase β-subunit	Acetate › polyketide
aflC	XM_002379910	*pksA*	Polyketide synthase	Acetate › polyketide
aflD	XM_002379908	*nor-1*	NOR reductase	NOR › AVN
aflE	XM_002379901	*norA*	NOR dehydrogenase	NOR › AVN
aflG	XM_002379896	*avnA*	CYP450 monooxygenase	AVN › HAVN
aflH	XM_002379903	*adhA*	Alcohol dehydrogenase	HAVN › AVF or AVNN
aflI	XM_002379893	*avfA*	CYP450 oxidase	AVF › VHA
aflJ	XM_002379902	*estA*	Esterase	VHA › VAL
aflK	XM_002379889	*vbs*	VERB synthase	VAL › VERB
aflL	XM_002379895	*verb*	VERB desaturase	VERB › VERA
aflM	XM_002379900	*ver-1*	Ketoreductase	VERA › DMST
aflN	XM_002379898	*verA*	Monooxygenase	VERA › DMST
aflO	XM_002379892	*omtB*	*O*-methyltransferase B	DMST › ST<br>DHDMST › DHST
aflP	DQ176782	*omtA*	*O*-methyltransferase A	ST › OMST<br>DHST › DHOMST
aflQ	XM_002379890	*ordA*	Oxidoreductase	OMST › AFB1, AFG1<br>DHOMST › AFB2, AFG2
aflR	XM_002379905	*apa-2*	Transcription activator	Pathway regulator
aflS	XM_002379904	UN[b]	Transcription enhancer	Pathway regulator

[a] Gene accession number in Gen-Bank NCBI databases.

[b] Unnamed gene

Table S2 Primers used for the transcriptional analysis of AF-biosynthesis cluster genes.

Gene	Primers name	Nucleotide sequence (5'-3')	Primer position	Amplicon (bp)
fas-α	*fasαF*	CAACGCCAACGCTATTCGAG	537- 556	180
	fasαR	GTAATGCCACACGATTCGGC	697- 716	
fas-β	*fasβF*	ATCCACTCGACATCATCGCC	2468- 2487	115
	fasβR	TTGATGTCACGTCGGCTGAA	2563-2582	
pksA	*pksAF*	TAGTGTGCCTCTGCCAGTTG	254- 273	107
	pksAR	GGAACCCATGCAGAATCCCA	341- 360	
nor-1	*nor1F*	GCATCGGACGAGGTCTCATT	158-177	170
	nor1R	CTGGGCATCAGTTTCCGAGT	308-327	
norA	*norAF*	TTGGTACTGAGCGAGGAGGA	940-959	159
	norAR	TTCTAGCCGAGTGTTGCAGG	1079-1098	
avnA	*avnAF*	ATCGACGACTGTTGGCCTTT	386-405	188
	avnAR	CGAGTCTCCAAAAGCGAGGT	554-573	
adhA	*adhAF*	TCTAGAGACGGGGCAGAACA	147-166	172
	adhAR	TGCAAAGGAGACACCTGCAA	299-318	
avfA	*avfAF*	AGTACCGGCCTTCGTTCATC	411-430	177
	avfAR	AGTCTGTAGCCCGTTGGTTG	568-587	
estA	*estAF*	ACGCTACGAGATGATGCCAG	766-785	150
	estAR	TCCCCCGAAGAAAGTCCTCT	896-915	
vbs	*vbsF*	CCGCTCTGATGACTCCCTTC	1509-1528	158
	vbsR	GTCCGATGCAACAATCTCGC	1647-1666	
verb	*verbF*	GATGCTCAATAACGCTGCCG	963-982	186
	verbR	GTAAGGTACGGCAGATGCGA	1129-1148	
ver-1	*ver1F*	TGGTGAACTACGCCCATTCC	110-129	137
	ver1R	CACCGTCTCCGCCATTAACT	227-246	
verA	*verAF*	CCTCAGCAGCCACCCAAATA	290-309	128
	verAR	CCGCCACTTCTTCCAAGTCT	398-417	
omtB	*omtBF*	GCAAACGGCAAATTCAGGGT	71-90	173
	omtBR	CGCTAGAGTTATCGGCGTGT	224-243	
omtA	*omtAF*	ATGTGACGAAGTGATGCGGT	294-313	156
	omtAR	CTCGCATTTCAGCTGCGTTC	430-449	
ordA	*ordAF*	ATTTGTGTTCGGCTTTGGGC	1524-1543	107
	ordAR	TGGGCGAGATGAAGAAGCAG	1611-163	
apa-2	*apa2F*	CGCTATTGCTGCTTTTCGCT	691-710	158
	apa2R	GCATCGGTAGCCCTCTTGTT	829-848	
TE[a]	*TEF*	AGGCTTTCTTTGTGAGCCGT	357-376	132
	TER	ATAGCTGATGCTGACGGAGC	469-488	

[a] TE: Transcriptional Enhancer encoding gene

Table S3 Primers used for cloning, silencing, mutagenesis, deletion and PCR-fusion constructions. Under-lined nucleotides indicate *BamHI*, *HindIII* and *SalI* restriction sites, respectively. Modified codon for a punctual mutagenesis is indicated in bold.

Gene/region	Primer name	Nucleotide sequence (5'-3')
AfPXG	AfPXGF	GGGGATCCATGCCTTCCAAAGTAAACATCG
	AfPXGR	GGAAGCTTTTACGAATATATCTTTTCTCCT
AfPXG	siRNAPXGF	UGGGCCAAUAAGGUCCGGGUU
	siRNAPXGR	CCCGGACCUUAUUGGCCCAUU
AfPXG/His85	H85/VF	GGGTCGACTACACCCCTCTCCAGCAG**GTC**GTCCTATTCTGGG
	H85/VR	GGGTCGACACGGTCCCAGAATAGGAC**GAC**CTGCTGGAGAGG
AfPXG/D	D126-140F	GGGTCGACCTGTGGTTTCGAGTTTAT
	D126-140R	GGGTCGACTTATAACTACTAGTTGTGCCG
5'-UTRF	P1	AAGTTCGGTTAAGTATGATATCAA
5'-UTR$_{Hyg}$R	P2	GAGTTCAGGCTTTTTCATAAACTATTAAATAACTCA
3'-UTR$_{Hyg}$F	P3	CCGAGGGCAAAGAAATAGTAAAGAGGCGGTTGATAC
3'-UTRR	P4	TCTTTGTACAGCGGTTACCAT
5'-UTR$_{Hyg}$F	P5	TGAGTTATTAATTAGTTTATGAAAAAGCCTGAACTC
3'-UTR$_{Hyg}$R	P6	AACTATGTATCAACCGCCCTATTTCTTTGCCCTCGG
Adh1ProF	P7	AAGCTTGACGTTTGACAGGGAA
Adh1Pro$_{gfp}$R	P8	CAGCTCCTCGCCCTTGCTCATTTGACTTTGGGATCTTGTGTTG
Gfp$_{Adh1pro}$F	P9	CAACACAAGATCCCAAAGTCAGTGAGCAAGGGCGAGGAGCTG
Gfp$_{AfPXG}$R	P10	CTTGTACAGCTCGTCCATGCCTACGGAAGGTTTCATTTGTA
AfPXG$_{gfp}$F	P11	GGCATGGACGAGCTGTACTTGATGCCTTCCAAAGTAAACAT
AfPXGR	P12	TTACGAATATATCTTTTCTCCT
5'-UTR$_{Adh1pro}$R	P13	GAGTTCAGGCTTTTTTTCCCTGTCAAACGTCAAGCTT
5'-UTR$_{Adh1pro}$F	P14	TGAGTTATTAATTAGTTTAAGCTTGACGTTTGACAGGGAA
3'-UTR$_{AfPXG}$R	P15	AACTATGTATCAACCGCCCTTTACGAATATATCTTTTCTCCT
3'-UTR$_{AfPXG}$F	P16	GGAGAAAAGATATATTCGTAATAAAGAGGCGGTTGATAC
3'-UTR$_{Ole1}$R	P17	AACTATGTATCAACCGCCCTTTAAGTAGTGTGCTGGCCACCA
3'-UTR$_{Ole1}$F	P18	TGGTGGCCAGCACACTACTTAAATAAAGAGGCGGTTGATAC

Table S4. NCBI accession number and physiological characteristics.

Species Name	Clo isoform	NCBI Accession No	Order Locus (gene)	Amino acids	Major CLO domains	MW (kDa)	Isoelectric point (pl)	Exons	Introns
Aspergillus flavus	AflCLO1	XP_002382486	AFLA_002850	290	77-261	32.81	5.53	4	3
Erysiphe necator	EnCLO1	KHJ32187	EV44_g5777	244	66-234	27.97	6.76	3	2
Neurospora crassa	NcCLO1	XP_958990	NCU09043	299	75-244	34.19	5.18	3	2
Magnaporthe oryzae	MoCLO1	XP_003708880	MGG_02110	265	84-251	30.4	5.42	3	2
Beauveria bassiana	BbCLO1	XP_008600624	BBA_07305	254	77-244	29.20	5.76	5	5
Ustilago maydis	UmCLO1	XP_011389113	UMAG_02753	252	58-234	28.77	7.09	3	2
Rhodotorula toruloides	RtCLO1	XP_016271628	RHTO_03428	345	104-272	39.38	5.85	5	4
Gonapodya prolifera	GprCLO1	KXS22162	M427DRAFT_106558	258	56-258	26.63	7.02	9	8
Rhizophagus irregularis	RiCLO1	ESA08389	GLOINDRAFT_349121	316	122-290	36.14	6.92	6	5
Allomyces macrogymus	AmaCLO1	KNE62883	AMAG_08061	378	126-298	41.55	6.14	4	3
Rozella allomycis	RaCLO1	EPZ32993	O9G_003724	162	1-153	19.12	6.43	4	3
